# Environment-responsive dendrobium polysaccharide hydrogel embedding manganese microsphere as a post-operative adjuvant to boost cascaded immune cycle against melanoma

**DOI:** 10.7150/thno.94354

**Published:** 2024-06-17

**Authors:** Nan Gao, Yiran Huang, Shisuo Jing, Meng Zhang, Ergang Liu, Lu Qiu, Jing Huang, Bahtiyor Muhitdinov, Yongzhuo Huang

**Affiliations:** 1 School of Pharmacy, Guizhou Medical University, Guizhou 561113, China; 2 Zhongshan Institute for Drug Discovery, SIMM, CAS, Zhongshan 528400, China; 3 Department of Pharmacy, Women's Hospital, Zhejiang University School of Medicine, Hangzhou 310006, China; 4 Artemisinin Research Center, Guangzhou University of Chinese Medicine, Guangzhou 510450, China; 5 Institute of Bioorganic Chemistry, Uzbekistan Academy of Sciences, Tashkent 100125, Uzbekistan; 6 Shanghai Institute of Materia Medica, Chinese Academy of Sciences, Shanghai 201203, China; 7 NMPA Key Laboratory for Quality Research and Evaluation of Pharmaceutical Excipients, Shanghai 201203, China

**Keywords:** post-operative implantation, dendrobium hydrogel, manganese pectin microspheres, cancer metalloimmunotherapy, tumor recurrence, metastasis

## Abstract

**Rationale:** Surgical resection is a primary treatment for solid tumors, but high rates of tumor recurrence and metastasis post-surgery present significant challenges. Manganese (Mn^2+^), known to enhance dendritic cell-mediated cancer immunotherapy by activating the cGAS-STING pathway, has potential in post-operative cancer management. However, achieving prolonged and localized delivery of Mn^2+^ to stimulate immune responses without systemic toxicity remains a challenge.

**Methods:** We developed a post-operative microenvironment-responsive dendrobium polysaccharide hydrogel embedded with Mn^2+^-pectin microspheres (MnP@DOP-Gel). This hydrogel system releases Mn^2+^-pectin microspheres (MnP) in response to ROS, and MnP shows a dual effect *in vitro*: promoting immunogenic cell death and activating immune cells (dendritic cells and macrophages). The efficacy of MnP@DOP-Gel as a post-surgical treatment and its potential for immune activation were assessed in both subcutaneous and metastatic melanoma models in mice, exploring its synergistic effect with anti-PD1 antibody.

**Result:** MnP@DOP-Gel exhibited ROS-responsive release of MnP, which could exert dual effects by inducing immunogenic cell death of tumor cells and activating dendritic cells and macrophages to initiate a cascade of anti-tumor immune responses. *In vivo* experiments showed that the implanted MnP@DOP-Gel significantly inhibited residual tumor growth and metastasis. Moreover, the combination of MnP@DOP-Gel and anti-PD1 antibody displayed superior therapeutic potency in preventing either metastasis or abscopal brain tumor growth.

**Conclusions:** MnP@DOP-Gel represents a promising drug-free strategy for cancer post-operative management. Utilizing this Mn^2+^-embedding and ROS-responsive delivery system, it regulates surgery-induced immune responses and promotes sustained anti-tumor responses, potentially increasing the effectiveness of surgical cancer treatments.

## Introduction

Surgery is a primary option for solid tumor treatment. In 2015, more than 80% of newly diagnosed cancers (15.2 million) required surgical resection 1, and the number of new cases with surgical indications was continually increasing 2. However, physical resection often results in immune suppression, which persists for weeks or even months as controlled by the healing process in the surgical wound 3-5. This weakens the immune system and promotes tumor growth at both the primary and metastatic sites 4, 6. Reportedly, 20-66% of cancer patients experienced post-surgical recurrence and metastasis 7, 8, which is also a major contributor to cancer mortality 4, 9. Therefore, transforming the tumor microenvironment (TME) from the pro-tumor to the anti-tumor type is crucial in cancer post-operative management 10-12.

Transition metal ions, such as Cu^2+^, Fe^2+/3+^, Mn^2+^, and Co^2+^, are essential parts of metalloproteins and play various biological functions 13. Recently, there has been a growing interest in the roles of metal ions in immune sensing and regulation, leading to a new research field called “metalloimmunology”. As a case in point, Mn^2+^ plays a crucial role in regulating the host's immune system 14, e.g., Mn^2+^ can promote dendritic cell (DC)-mediated cancer immunotherapy by stimulating the cGAS-STING (cyclic guanosine monophosphate-adenosine monophosphate synthase-stimulator of interferon genes) pathway 15. Notably, there are advantages of Mn^2+^ for clinical application, such as lower oxidation potential than Fe^3+^ and less toxic than Cu^2+^ and Co^2+^, thus serving as a promising agent for immunotherapy 16-18. However, the recommended daily intake of Mn^2+^ for human adults is only 1.8-2.3 mg 16, and the normal blood concentration of Mn^2+^ in humans is below 1 µM 19. In comparison, robust DC responses are elicited by concentrated Mn^2+^ of above 200 µM or even 2 mM 20. Systemic exposure to a high concentration of Mn^2+^ leads to neurotoxicity 21, 22, and thereby, it imposes a formidable challenge to achieve sufficient Mn^2+^ levels in the tumors to activate local anticancer immunity while restricting side toxicities.

To address these two issues mentioned above, we developed a drug-free, post-surgical implantation strategy using polysaccharide hydrogel embedding Mn^2+^-loaded microspheres for initiating metalloimmunity and preventing tumor recurrence. Specifically, Mn^2+^ was chelated with pectin to form Mn^2+^-pectin microspheres (MnP), which were subsequently incorporated into a dendrobium polysaccharide (DOP) hydrogel (termed MnP@DOP-Gel). MnP@DOP-Gel utilized as the post-surgical implant is designed to respond to dynamic changes in the TME after surgery. Notably, surgery can cause significant inflammation and oxidative stress (e.g., a marked increase in reactive oxygen species (ROS) level) in surgical sites 23. High oxidative stress is linked with poor prognosis in cancer patients undergoing surgery 24. ROS-scavenging gels are often applied against postsurgical cancer recurrence and metastasis 25. DOP could reduce oxidative stress-induced injury 26, thus benefiting from alleviating acute post-surgical inflammation. It is expected that DOP-Gel can take action of slow degradation responsive to the post-surgical environment and the subsequently released MnP microspheres could promote Mn^2+^-mediated metalloimmunity in situ.

In this work, we assessed the post-surgical treatment efficacy of MnP@DOP-Gel in a murine melanoma model and investigated the synergistic effects of MnP@DOP-Gel and anti-PD1 antibody (aPD1) for combination therapy.

## Results

### Preparation and characterization of MnP@DOP-Gel

**Figure 1:**
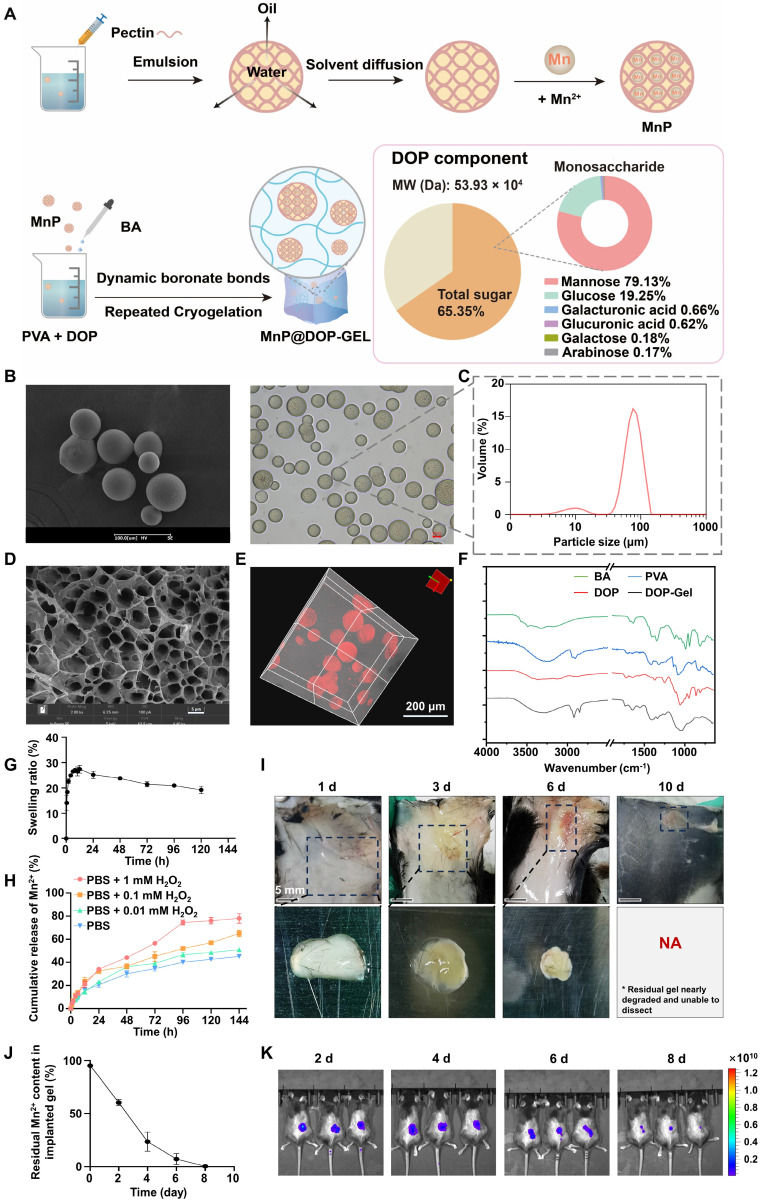
Preparation and characterization of MnP@DOP-Gel. A, Schematic illustration of MnP@DOP-Gel preparation and DOP characterization. B, Microscopic images of MnPs by SEM (scale bar, 100 μm) and inverted microscope (scale bar, 10 μm). C, Size distribution of MnP determined by dynamic light scattering analysis. D, SEM image (scale bar, 5 μm) of DOP-Gel. E, Confocal image of MnP@DOP-Gel (MnPs labeled by Nile red; scale bar, 200 μm). F, FTIR of DOP-Gel. G, Swelling ratio of DOP-Gel. H, *In vitro* release of Mn^2+^ from MnP@DOP-Gel under different H_2_O_2_ concentrations. I, *In vivo* degradation of subcutaneously injected MnP@DOP-Gel in normal mice (scale bar, 5 mm). J, In situ release of Mn^2+^, indicated by the residual Mn^2+^ content in MnP@DOP-Gel at 2 d, 4 d, 6 d, and 8 d post-implantation. K, Fluorescence images of mice 2 d, 4 d, 6 d, and 8 d after post-operative implantation of MnP@DOP-Gel.

As a natural polysaccharide rich in hydroxyl and glucuronic acid groups, pectin can efficiently chelate multivalent metal ions and form reversible metal-polymer complexes 27. Mn^2+^-crosslinked pectin microspheres (MnP) were prepared by a solvent diffusion method (Figure 1A). The thus-obtained MnP had a high Mn^2+^-loading capacity of 35% and manifested an average size of 70 μm (Figure 1B-C). The saccharide purity, molecular weight, and composition of dendrobium polysaccharide (DOP) were determined and the results are shown in Figure 1A. MnP was further incorporated into DOP hydrogel cross-linked by borate bonds, which were further condensed through repeated congelation (Figure 1A). The thus-formed DOP-Gel manifested typical Fourier transform infrared (FTIR) signals at 1340 cm^-1^ (B-O bond) and 1370 cm^-1^ (C-O bond), confirming the formation of borate bonds in the DOP-Gel (Figure 1F). Scanning electron microscopy (SEM) revealed the porous structure of DOP-Gel (Figure 1D). Confocal laser scanning microscopy (CLSM) observation revealed that MnPs (labeled by Nile red) were evenly dispersed in the gel matrix (Figure 1E).

The biocompatibility of this hydrogel was evaluated by incubating human umbilical vein endothelial cells (HUVEC) with the leachates of DOP-Gel, and no discernible cytotoxicity was observed (Figure S1). The time-dependent swelling profiles of DOP-Gel are shown in Figure 1G. The hydrogel swelled within a time frame of 6 h, which is helpful for absorbing inflammatory exudates by serving as surgical implants. There was an accelerated release of Mn^2+^ from the gel responsive to the elevating H_2_O_2_ levels (Figure 1H). It should be pointed out that this release test was conducted in phosphate-buffered saline (PBS) containing 0.01-1 mM H_2_O_2_, with neutral pH measured to be around 7.4, thereby indicating the responsiveness was dependent on H_2_O_2_ concentrations, not relying on pH.

Given that surgical stress could induce reactive oxygen species (ROS) in the wounds 23, 28, we further assessed the *in vivo* degradation behavior of the implanting MnP@DOP-Gel by responding to post-surgical stress. DOP-Gel loaded with Nile red-labeled MnP was injected at the resection site of the subcutaneous tumor. By *in vivo* imaging, a persistent fluorescence with decreasing intensity over time was observed at the implanting site (Figure 1K), indicating sustained release of Nile red from the encapsulated MnP. Of note, the degradation of the implanting MnP@DOP-Gel in the resected tumor was faster (within 8 days, Figure 1K) than in the subcutaneous site without tumor lesions (in 10 days, Figure 1I). It suggested that degradation was accelerated in the surgical environment due to the increased ROS. Consequently, around 90% of Mn^2+^ were released from the implants 6 days post-surgery (Figure 1J). More importantly, blood Mn^2+^ levels remained within the normal physiological range (Table S1), indicating the safety of local administration of a high dose of MnP@DOP-Gel (30 mg/kg). No significant change in blood Mn level suggested manganese was mainly taken locally, thus without systemic exposure concern.

### Induction of ICD of melanoma cells and initiation of antitumor immune responses by MnP

MnP treatment activated the cyclic guanosine monophosphate-adenosine monophosphate synthase-stimulator of interferon genes (cGAS-STING) pathway in murine melanoma cells (B16-F10), as confirmed by real-time quantitative polymerase chain reaction (RT-qPCR) and western blotting (WB) analysis. The expression of the related factors, including tank binding kinase 1 (TBK1), cyclic GMP-AMP synthase (cGAS), and stimulator of interferon genes (STING), was upregulated (Figure S2). MnP treatment also upregulated interferon-1 beta (IFN-1β) expression (Figure S3). It is recognized that cGAS-STING/type I IFN plays a key role in promoting immunogenic cell death (ICD) and apoptosis 29.

**Figure 2:**
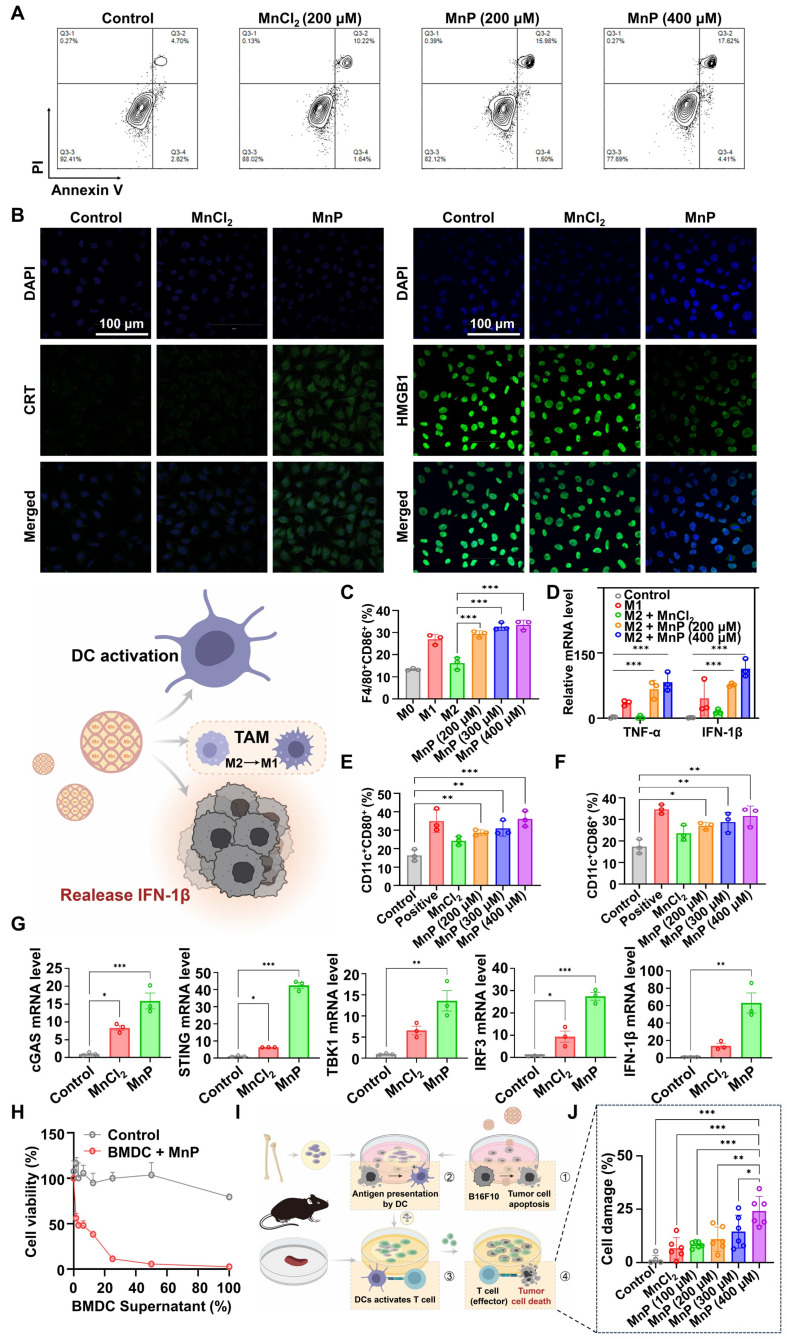
*In vitro* assessment of MnP orchestrated tumor-specific immune responses. A, MnP-induced apoptosis in B16-F10 cells (MnCl_2_ = 200 μM, MnP = 200 μM, 400 μM). B, CRT and HMGB1 expression were observed by confocal microscopy (MnCl_2_ = 200 μM, MnP = 200 μM; scale bar, 100 μm). C, Proportional M1 type cells in MnP-treated M2 macrophages. D, mRNA levels of IFN-1β and TNF-α. E and F, Maturation of DCs (CD11c^+^CD80^+^, CD11c^+^CD86^+^) induced by MnP. G, mRNA levels of STING-associated biomarkers (cGAS, STING, TBK1, IRF3, and IFN-1β) in BMDCs. H, Cytotoxic effect of supernatants from MnP-treated BMDCs on B16-F10 cells. I, Schematic illustration of MnP orchestrating tumor cell apoptosis, DC maturation, antigen presentation, CD8^+^ T cells activation, and CD8^+^ T cell-mediated tumor-specific killing. J, Specific killing of B16-F10 cells by T cells activated by BMDCs. *, P < 0.05; **, P < 0.01; ***, P < 0.001.

MnP induced a higher apoptosis rate (17.5%) in B16-F10 cells compared to free Mn^2+^ (11.9%) at a Mn^2+^ concentration of 200 µM (Figure 2A). Of note, the increased IFN-1β expression may further promote the apoptosis of tumor cells in a self-motivated manner 30, 31. Besides apoptosis, a significant increase in calreticulin (CRT, the “eat-me” signal for the immune cells) was observed in MnP-treated B16-F10 cells by both flow cytometry (Figure S5) and immunofluorescence imaging (Figure 2B, Figure S4). Meanwhile, MnP treatment also caused a substantial increase in ATP levels (“find-me” signal) in the B16-F10 cells (Figure S6). Interestingly, the nuclear high mobility group box-1 protein (HMGB1) was inversely reduced (Figure 2B, Figure S4-5) in contrast to increased cytosol HMGB1 after MnP treatment. The result suggested the translocation of HMGB1 from nuclei to the cytosol, which was a crucial event for the damage-associated molecular pattern (DAMP) and immune stimulation 32. Therefore, MnP significantly promoted tumor apoptosis and ICD, facilitating the activation of dendritic cells (DCs) and initiation of antitumor immune responses 33, 34.

Activating tumor-associated macrophages (TAMs) and DCs are useful strategies for tumor immunotherapy by remodeling the tumor immunosuppressive microenvironment (TIME) 35. TAMs are generally classified as anti-tumor M1 type and pro-tumor M2 type, with the latter being the dominating cells in the tumor microenvironment (TME) and a key immunosuppressor 9. Repolarization from the M2 to M1 phenotype is an effective means to induce anticancer immunity 36, 37. We thus analyzed the potential effect of MnP on M2-type bone marrow-derived macrophages (BMDMs), finding that the population of M1 phenotype was significantly expanded (34.8%) as compared with control (13.2%) (Figure S7). Doubling the concentration of MnP from 200 µM to 400 µM showed a dose-dependent promotion of macrophage repolarization from the M2 to the M1 phenotype (Figure 2C). Accordingly, there was a more than 10-fold increase in the mRNA levels of antitumoral cytokines (TNF-α and IFN-1β) in MnP-treated macrophages compared to free Mn^2+^ (Figure 2D), manifesting a superior effect of MnP in priming innate antitumor responses, but without inducing obvious cytotoxicity (Figure S8).

The adjuvant effect of MnP was evaluated on bone marrow-derived dendritic cells (BMDCs). Cytotoxicity assay demonstrated that MnP did not significantly inhibit DC proliferation (Figure S8). Meanwhile, the population of the matured BMDCs (CD80^+^CD86^+^) was increased in a dose-dependent manner, suggesting the potent DC activation effect of MnP (Figure 2E-F, Figure S9). The cGAS-STING pathway in DCs was activated by MnP treatment, as reflected by the elevated mRNA expression levels of cGAS, STING, TBK1, and interferon regulatory factor 3 (IRF3) (Figure 2G). As a result, IFN-I was upregulated (Figure 2G), which is an apoptosis inducer and immune stimulator 34.

The increased IFN-I could thus synergize with MnP-induced apoptosis as well as promote antitumor effects of macrophages and DCs. As evidence, the culture medium supernatant of MnP-treated DCs showed an inhibitory effect on the growth of tumor cells (Figure 2H). We also assessed the cytotoxic effects of CD8^+^ T cells primed by the activated BMDCs that were induced by the MnP-treated dying B16-F10 tumor cells (Figure 2I). The results showed that co-culture with supernatant from MnP-treated tumor cells promoted BMDC maturation (Figure S10), which then induced the cytotoxic effects of T cells against tumor cells, resulting in dose-dependent cell killing (Figure 2J). Of note, the active T cells primed by the MnP-pretreated B16-F10 cells specifically killed the same type of cancer cells (i.e., B16-F10), but not other types (e.g., Lewis lung carcinoma (LLC) cells), suggesting successful presentation of melanoma-specific antigens by BMDCs to T cells (Figure S11).

### *In vivo* therapy efficacy of MnP@DOP-Gel

**Figure 3:**
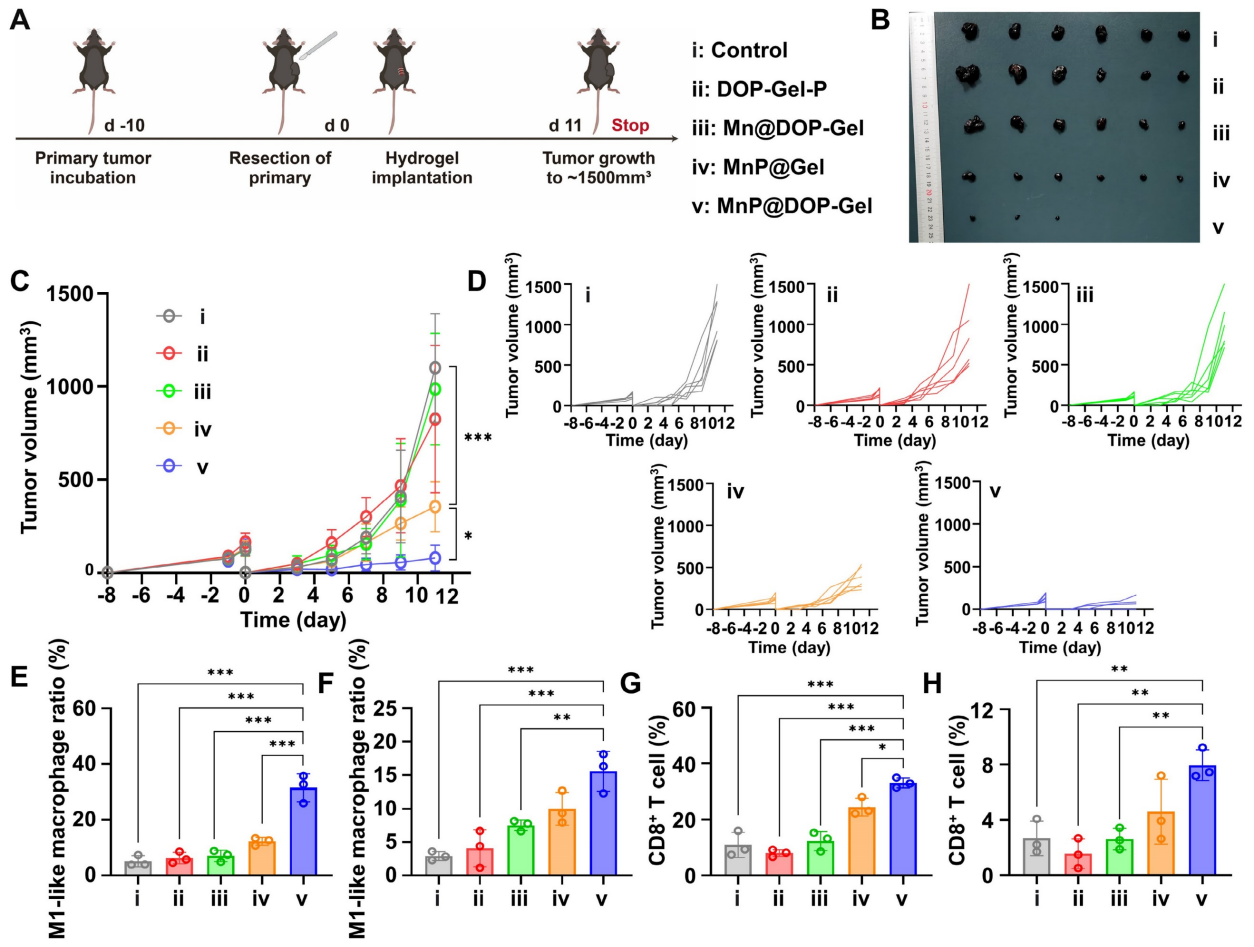
***In vivo* therapeutic assessment of implanting MnP@DOP-Gel on subcutaneous B16-F10 tumor model.** A, Experimental schedule of post-operative treatment. B, Tumor images at experimental endpoint. C, Tumor growth curves. D, Tumor growth curves of each mouse (n = 6). M1-type macrophage proportions in lymph nodes (E) and spleen (F). CD8^+^ T cell proportions in the spleen (G) and reoccurring tumors (H). *, P < 0.05; **, P < 0.01; ***, P < 0.001.

The therapeutic efficacy of post-operative implanting MnP@DOP-Gel was assessed in a subcutaneous melanoma model. The growth of the residual tumors was monitored every other day (Figure 3A). The mice treated with free Mn^2+^-dispersed DOP-Gel showed a marginal inhibition against tumor growth, whereas MnP@Gel and Mn@DOP-Gel had a substantial efficacy. MnP@DOP-Gel demonstrated the most pronounced antitumor efficacy, resulting in a complete tumor regression rate of 50% (3/6). This outcome was likely attributable to the synergistic effect of DOP and MnP, benefiting from the gel structure for slow and controlled release of the embedded MnP. Consequently, up to 88.8% of tumor growth inhibition rate was achieved at the endpoint of the experiment (1500 mm^3^ for control, and 168 mm^3^ for MnP@DOP-Gel) (Figure 3B-D, Figure S12).

Immunological analysis of tumor-draining lymph node (TdLN) and splenic macrophage manifested a 6.5-fold (26% vs 4%) and 7.1-fold increase (16.3% vs 2.3%) of M1-type macrophage populations, respectively, in the MnP@DOP-Gel group compared to the control (Figure 3E-F, Figure S13). Meanwhile, MnP@DOP-Gel treatment also promoted T cell priming and activation, as evidenced by the increase in splenic CD8^+^ T cells and intratumoral infiltration of CD8^+^ T cells (Figure 3G-H, Figure S14). Safety assessment showed no obvious loss of body weight (Figure S15), and hematoxylin and eosin staining (H&E) examination of the major organs showed no pathological changes at the experimental endpoint (Figure S16).

### MnP@DOP-Gel improving the therapeutic efficacy of aPD1 antibody and preventing pulmonary metastasis

**Figure 4:**
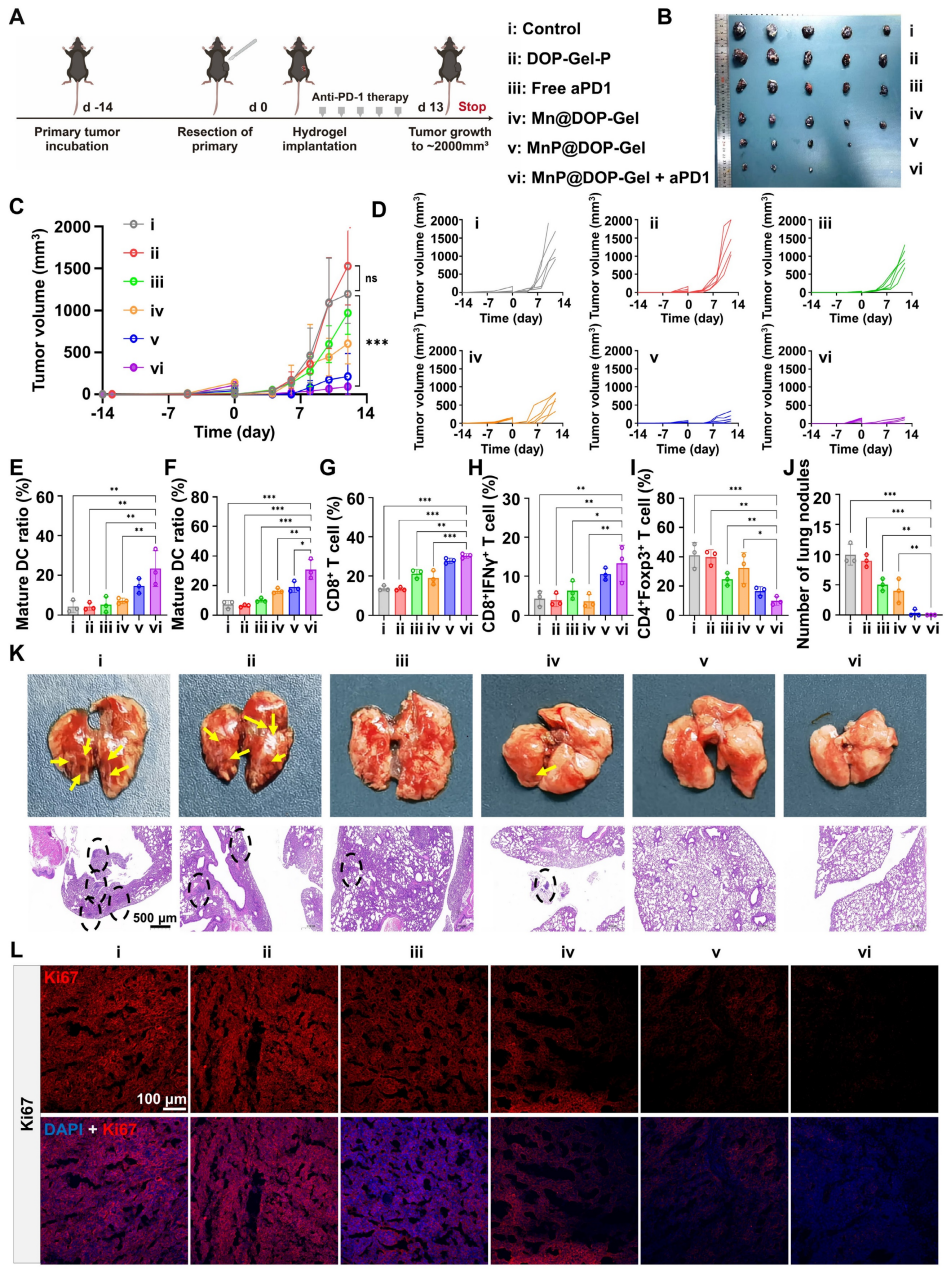
***In vivo* anti-metastasis assessment of implanting MnP@DOP-Gel in combination with aPD1.** A, Experimental schedule. B, Tumor images at experimental endpoint. C, Tumor growth curves. D, Tumor growth curves of each mouse (n = 5). Matured DC proportions in the reoccurring tumors (E) and lymph nodes (F). G, CD8^+^ T cell proportions in the spleen. H, Cytotoxic IFNγ^+^CD8^+^ T cell proportions in the spleen. I, Tregs in the spleen. J, Quantification of lung metastatic tumor nodules. K, Lung metastasis and H&E sections (scale bar, 500 μm). L, Ki67 staining in the tumors (scale bar, 100 μm). *, P < 0.05; **, P < 0.01; ***, P < 0.001; ns, nonsignificant.

As MnP@DOP-Gel demonstrated the adjuvant potency of boosting tumor-specific immunity, we further assessed the synergistic effect of MnP@DOP-Gel with the checkpoint blockade therapy of anti-PD1 antibody (aPD1). Following the same post-operative adjuvant procedures, aPD1 (10 mg/kg) was intraperitoneally given in combination with MnP@DOP-Gel (Figure 4A). Figure 4B-D shows a synergistic effect of MnP@DOP-Gel and aPD1, with a significantly higher efficient tumor inhibition rate (95.4%) compared to MnP@DOP-Gel alone (87.2%) (Figure S17). The combination of MnP@DOP-Gel and aPD1 also led to an increased proportion of mature DCs in reoccurring tumors and TdLN (Figure 4E-F, Figure S18). Additionally, there was an increase in cytotoxic T cells (CD8^+^ T cell & CD8^+^IFNγ^+^ T cells) in the spleen, alongside a reduction in the spleen's regulatory T cell (Treg) populations (Figure 4G-I, Figure S19). Accordingly, tumor metastasis to the lung was suppressed, and H&E staining showed no neoplastic nodules in lung specimens from the animals receiving MnP@DOP-Gel + aPD1 (Figure 4K). No neoplastic nodules were found in the MnP@DOP-gel + aPD1 group (Figure 4J). Meanwhile, the growth of the residual tumor cells as represented by Ki67 staining was also significantly suppressed in the MnP@DOP-Gel + aPD1 group, compared to other groups (Figure 4L, Figure S20), indicating the synergistic inhibition of tumor cell proliferation of MnP@DOP-Gel and free aPD1.

**Figure 5:**
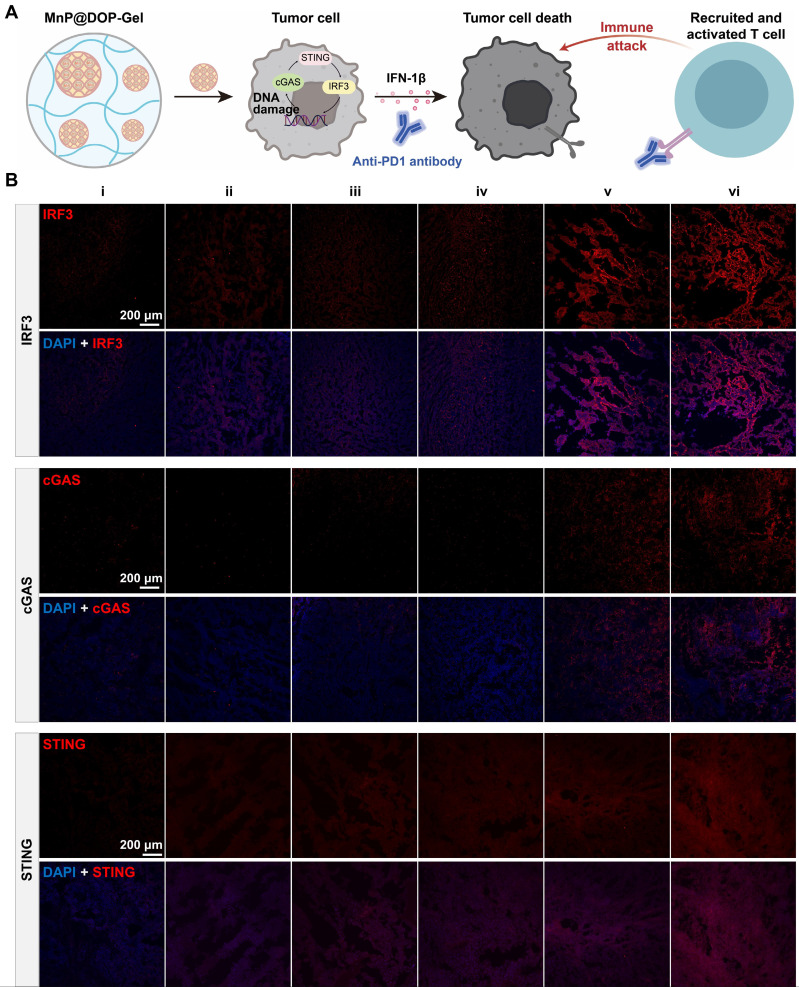
** Activation of cGAS-STING pathway in tumors.** A, Illustration of MnP@DOP-Gel combined with aPD1 for mediating T cell killing. B, Immunofluorescent staining of tumor sections of IRF3, cGAS, and STING (scale bar, 200 μm).

The therapeutic efficacy of aPD1 was not only affected by the expression of PD1 but also hampered by the immunosuppressive TME, and activation of the STING pathway by MnP@DOP-Gel could orchestrate a cascade of immune cycles against the remaining tumors (Figure 5A). The immunofluorescence staining results showed that STING biomarkers including IRF3, cGAS, and STING, were up-regulated in the MnP@DOP-Gel treated mice (Figure 5B, Figure S21). The activated STING pathway could promote anticancer immunity and enhance the apoptosis of tumor cells; this contributed to the increased therapeutic efficiency of aPD1.

### Combination therapy of MnP@DOP-Gel and aPD1 inhibiting abscopal brain tumor growth

**Figure 6:**
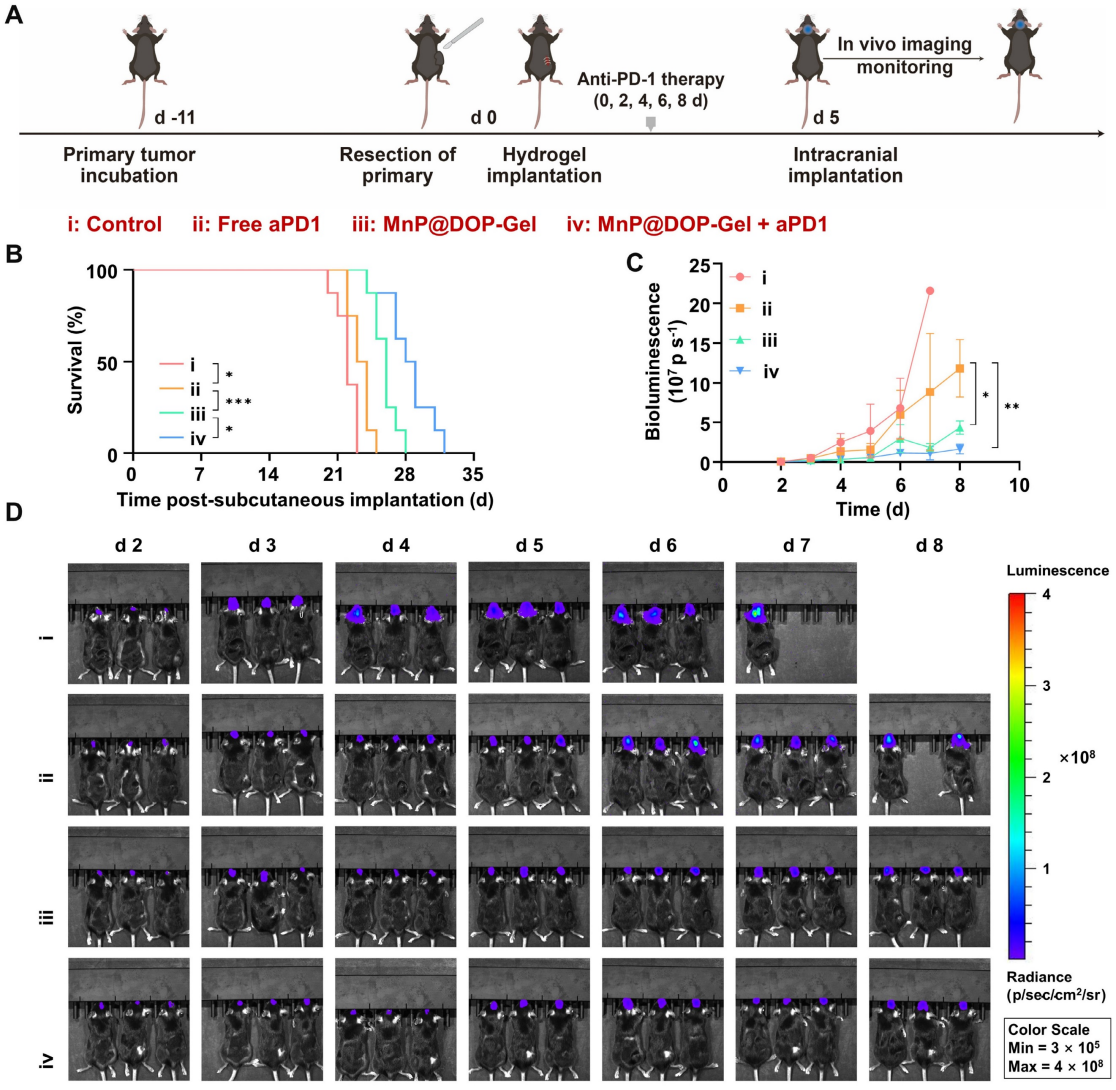
***In vivo* therapeutic assessment of implanting MnP@DOP-Gel in combination with aPD1 against an abscopal brain tumor.** A, Schedules of the experiment. B, Kaplan-Meier survival curves of the mice bearing brain tumors (n = 8). C, Tumor growth curves of brain tumors quantified by bioluminescent imaging. D, Bioluminescent images of mice (n = 3). *, P < 0.05; **, P < 0.01; ***, P < 0.001.

Melanoma accounts for the third prevalent cause of brain metastasis and the progression to brain metastasis leads to poor prognosis 38. Activated T cells can migrate to the brain and induce antitumor responses 39, 40, providing a potential strategy for inhibiting brain tumors. Following the same post-operative adjuvant procedures of the primary model of melanoma, a secondary tumor was inoculated by intracranial injection of luciferase-expressing B16-F10 cells (Figure 6A). The results revealed that the MnP@DOP-Gel + aPD1 showed the most pronounced effect in prolonging survival time (Figure 6B) and delaying melanoma growth (Figure 6C). The growth of brain tumors was monitored using *in vivo* imaging of the bio-luminescence activated in tumors (Figure 6D), also demonstrating the superior efficacy of MnP@DOP-Gel + aPD1. Therefore, it suggested that the local administration of MnP@DOP-Gel in the primary tumor site activated anticancer immunity that inhibited the abscopal brain tumor, synergizing the therapeutic effect of aPD1.

## Discussion

Surgery is the primary clinical treatment for cancer, and post-surgery intervention is crucial for preventing tumor recurrence and metastasis. However, postoperative adjuvant chemotherapy did not show positive benefits in patients at high risk for melanoma recurrence 41. Immune checkpoint inhibitors have also been clinically investigated for adjuvant immunotherapy for melanoma, showing benefits in recurrence-free survival, but not consistently for distant metastases-free survival or overall survival 42. Therefore, exploring new adjuvant strategies to improve the efficacy of surgical treatment is a pressing need in cancer therapy. MnP@DOP-Gel utilized the dual functions of Mn^2+^ in tumor cell-killing and immune cell activation, serving as a drug-free adjuvant to prime specific tumor immunity by inducing the ICD of residual tumor cells and immune cell activation. This implantable material avoided drug-related adverse effects caused by unwanted exposure and synergized the therapeutic potency of checkpoint blockade therapy. It provides an effective prophylactic strategy in cancer post-operative treatment and manifests good values for clinical translation.

Dendrobium has been used for anti-inflammation in traditional Chinese medicine. DOP, a key bioactive component of dendrobium, can inhibit oxidative stress and alleviate oxidative stress-induced damage 43, 44. These properties suggest that DOP may reduce oxidative stress-induced injury in the post-surgical environment. In this work, we utilized the antioxidant DOP to prepare environment-responsive hydrogel. Unlike conventional ROS-responsive materials that typically exhibited fast drug release 45, this DOP-Gel crosslinking by dynamic borate bonds is suitable for slow release by responding to post-operative pathology conditions with ROS overproduction. Recent research revealed that H_2_O_2_ promotes borate bond oxidation and results in hydrogel degradation 46. In addition, polysaccharides are sensitive to oxidative stress, leading to structural changes like glycosidic bond cleavage and depolymerization 47, 48. For instance, H_2_O_2_ facilitates the degradation of DOP 49.

By integrating MnP into the DOP hydrogel framework, MnP@DOP-Gel effectively healed post-surgical wounds and suppressed tumor recurrence. The relief of post-surgical stress and reconstitution of the vascular and collagen matrix is crucial for preventing tumor recurrence and metastasis 50, 51, which could be an action mechanism of DOP-Gel, thus synergizing with MnP and yielding enhanced antitumor efficacy compared to MnP@Gel alone.

Mn^2+^ is widely diffusible in the tissues and must be administered repeatedly to maintain a sustained immune activation. It is a challenge to specifically deliver manganese to the tumors while restricting unwanted systemic exposure and the potential side effects. To address this challenge, MnP@DOP-Gel was developed, which is a drug-free system and only contains dietary polysaccharides and manganese; both are biocompatible. In addition, local administration in surgical sites can benefit from maintaining sufficient therapeutic concentration in the tumor resection site but reducing systemic exposure of Mn^2+^, thus without safety concerns. The MnP@DOP-Gel's meticulously engineered structure allows for a progressive degradation over an 8-day period and for sustained release of the encapsulated MnP in response to surgical stress, thus averting burst release of Mn^2+^ and maintaining blood Mn^2+^ concentrations within safe limits throughout the duration of treatment. Such strategic delivery is substantiated by no significant body weight loss or discernible pathological changes in vital organs, underscoring the safety and biocompatibility of this system.

In the animal body, the released MnP could simultaneously exert a dual effect of inducing both tumor cell apoptosis and immune cell (DCs and macrophages) activation. The two processes mutually initiated an antitumoral immune cycle of in situ antigen production and presentation, which further stimulated T cell-mediated cell killing. The cohort engagement of DCs, macrophages, and T cells as well as tumor cells synergistically manifested combined antitumor immune responses.

## Conclusion

In this study, a ROS-responsive dendrobium polysaccharide hydrogel embedding manganese-pectin microspheres (MnP@DOP-Gel) was developed and used as an in-situ adjuvant for post-operative treatment of melanoma. The DOP hydrogel was formed by crosslinking via reversible borate ester bonds with PVA and borax, with environment-responsive degradation in the wounds and allowing for sustained release of embedded MnP and Mn^2+^. MnP was nontoxic to normal cells and was able to activate the cGAS-STING pathway and promote type I interferon production, thus inducing tumor cell ICD, M1 polarization, DC maturation, antigen presentation, and T cell-mediated tumor cell killing. It in turn promoted the antigen production and presentation process, thus orchestrating the cascaded antitumor immune cycle. MnP@DOP-Gel for post-surgical implantation significantly inhibited tumor recurrence and prevented lung metastasis. Furthermore, the implanted MnP@DOP-Gel yielded a synergistic effect with aPD1 on preventing either metastasis or abscopal brain tumor growth. MnP@DOP-Gel showed promise for post-operative management of cancer.

## Methods

### Materials and cells

Apple pectin (purity: > 64.5%) was obtained from Macklin Biochemical Co., Ltd. (Shanghai, China). Sodium tetraborate decahydrate (Borax) was obtained from the Guangzhou Chemical Reagent Factory (Guangzhou, China). PVA (alcoholysis degree: 98.0-99.0 mol%, viscosity: 54.0-66.0 mPa.s), and bovine serum albumin (BSA) were obtained from Aladdin Biochemical Technology Co., Ltd. (Shanghai, China). MnCl_2_ was purchased from Meryer Biochemical Technology Co., Ltd. (Shanghai, China). Trifluoroacetic acid (TFA), and methyl alcohol were purchased from ANPEL Laboratory Technologies Inc (Shanghai, China). Sodium hydroxide and sodium acetate trihydrate were purchased from Sigma-Aldrich (Saint Louis, USA). Annexin V-FITC/PI apoptosis detection kit was obtained from Vazyme Biotech Co., Ltd. (Nanjing, China). Gibo™ fetal bovine serum (FBS), RPMI 1640, Dulbecco's modified eagle's medium (DMEM), and TRIzol reagent were purchased from Gibco® Thermo Fisher Scientific Inc. (Waltham, USA). Antibodies (F4/80-FITC, IFN-γ-brilliant violet 510, Foxp3 brilliant violet 421, CD8a-PE, HMGB1-PE) were purchased from Biolegend (San Diego, USA). The antibodies (CD45-APC-Cy7, CD45-Alexa Fluor@700, CD45-PerCP-Cy5.5, CD3-Percp-Cy5.5, CD11c-FITC, CD80-PE, CD86-APC, CD4-APC, CD4-FITC, CD11b-Brilliant Violet 421, and CD86-PE-Cy7) were purchased from BD Pharmingen (San Diego, USA). Nile red, Hifair II 1st strand cDNA synthesis SuperMix and Hieff qPCR SYBR Green Master Mix (No Rox), Alexa Fluor 488 AffiniPure Goat Anti-Rabbit IgG (H+L), and 3-(4,5-dimethylthiazol-2-yr)-2,5-diphenyltetrazolium bromide (MTT) were purchased from Yeasen Biotechnology Co., Ltd (Shanghai, China). LDH cytotoxicity assay kit was purchased from Leagene Biotechnology Ltd. (Anhui, China). Primers were purchased from BGI (Shenzhen, China). Interferon-gamma (IFN-γ), recombinant murine M-CSF (macrophage colony-stimulating factor), recombinant murine GM-CSF (granulocyte-macrophage colony-stimulating factor), and murine IL-4 (interleukin-4) were purchased from PeproTech Inc. (Cranbury, USA). Collagenase IV, hyaluronidase grade I, and polyvinylidene fluoride were obtained from Biosharp (Hefei, China). Trypsin-EDTA (0.25%), calreticulin rabbit mAb, HMGB1 rabbit mAb, one-step PAGE gel fast preparation kit, and quickblock™ blocking buffer were purchased from Beyotime Biotechnology Co., Ltd. (Shanghai, China). DAPI Fluoromount-G® was obtained from Southern Biotechnology Associates, Inc. (Birmingham, USA). Anti-STING (anti-TMEM173) antibodies were obtained from Proteintech (Wuhan, China). Anti-Ki67 antibodies, anti-IRF3 antibodies, and anti-cGAS antibodies were obtained from ABclonal Technology (Wuhan, China). Potassium (S)-2-(6-hydroxybenzo[d]thiazol-2-yl)-4,5-dihydrothiazole-4-carboxylate was obtained from Bide Pharmatech Co., Ltd. (Shanghai, China).

HUVEC and B16-F10 were obtained from the National Collection of authenticated cell cultures (Shanghai, China). LLC cells were obtained from Procell (Wuhan, China).

### Animals

All the animal experimental procedures were complied with the institutional ethical guidelines and approved by Zhongshan Institute for Drug Discovery. Female C57BL/6 mice (6-8 weeks old) were purchased from BesTest Bio-Tech Co., Ltd. (Zhuhai, China). All animals were housed at a specific pathogen-free care facility with free access to food and water, under a 12 h light-dark cycle.

### Preparation and characterization of MnP

The pectin/Mn^2+^ microspheres (MnP) were prepared following an emulsification-solvent diffusion method 27. Briefly, 3 mL ethanol was added into 17 mL pectin aqueous solution (1.5%, w/v) and stirred for 15 min (600 rpm). The mixture was then dropwise added to a 4 L organic phase composed of 50% ethyl acetate and 50% dichloromethane under stirring (750 rpm) for 20 min. The thus-formed pectin microspheres were collected as precipitates, which were subjected to air-drying and sieving by a 70-micron filter to remove aggregates. Mn^2+^ loading was carried out by immersing 150 mg pectin microspheres in 30 mL MnCl_2_ solution (10%, w/v) and incubating for 3 h, which was followed by centrifugal collection and washing with water. The salt immersion-centrifugation-washing procedure was repeated three times before freeze-drying.

The loading efficiency of Mn^2+^ was determined by inductively coupled plasma optical emission spectrometry (ICP-MS, iCAP-TQ, Thermo Fisher, Waltham, USA). Morphologies of MnPs were assessed by scanning electron microscopy (SEM, TESCAN MIRA LMS, Tescan, Brno, Czech), and the size was measured by a laser diffraction analysis instrument (Malvern Mastersizer 3000, Malvern, Worcestershire, UK).

### Preparation of DOP

The polysaccharide of dendrobium (DOP) was obtained following the two-step protocol of water extraction and alcohol precipitation 52. Briefly, fresh stems of dendrobium were first pretreated by peeling, cutting into small pieces, and triple washing with water. The stems (100 g) were mixed with 2,000 mL water and pulverized by a household homogenizer. The dendrobium pulp was then centrifuged at 2,000*×g* for 20 min to remove large tissue fragments, followed by centrifugation at 12,000*×g* for 60 min to remove fine debris from plant cells. The supernatants were collected and precipitated by excess 95% ethanol (v/v, 4 times the volume of the dendrobium syrups), followed by washing with 80% ethanol (v/v) 2 times. The precipitates were crude polysaccharides, which were weighed and redissolved in distilled water (v/v, 1%). A mixture of chloroform-n-butanol (4:1, v/v), accounting for a quarter of the volume of the polysaccharide solution, was added and mixed, followed by centrifugation (3,000 rpm, 20 min) to remove water-soluble proteins. The aqueous solutions were subjected to dialysis (8,000-12,000 Da) to remove oligosaccharide species. The remaining dialysate was freeze-dried to obtain DOP.

### Molecular characterization and total and monosaccharide analysis of DOP

The molecular weight of DOP was determined using high-performance gel permeation chromatography (HPGPC) (1260 Infinity II, Agilent, California, USA). The analysis was carried out at a controlled column temperature of 40.0 ± 0.1°C. NaCl solution (0.2 M) was used as the mobile phase at a flowing rate of 0.6 mL/min. Samples were prepared by dissolving to a concentration of 0.2% (w/v) using the mobile phase solution. Following centrifugation, the supernatant was collected for analysis (chromatography columns: TSK-Gel G4000PWXL column). Each sample was injected in a volume of 5 μL per run.

To measure the total polysaccharide content, 1 mL aqueous DOP solution (0.1 mg/mL) was mixed with 0.05 mL phenol solution (80%, distilled water as solvent) and 5 mL H_2_SO_4_ (98.3%). The mixtures were allowed to stand for 30 minutes. OD_490_ of the sample was measured using a UV spectrophotometer (TU-1900, Beijing, China), and the saccharide content was calculated using the serial concentrations of glucose solutions as the standard.

For monosaccharide analysis, 5 mg of DOP was dissolved in 1 mL of 2 M Trifluoroacetic acid (TFA) and allowed to hydrolyze at 121°C for 2 h. The hydrolysates were then air-dried by nitrogen flow, washed with 1 mL methanol 3 times, and redried to remove methanol. The residues were redissolved in 1 mL deionized water, filtered, and then analyzed by high-performance ion chromatography (HPIC) with a flow rate of 0.5 mL/min and an injection volume of 5 μL (Dionex ICS 5000+ with electrochemical detector, Thermo Fisher Scientific, Waltham, USA).

### Preparation and characterization of MnP@DOP-Gel

DOP (60 mg) was dissolved in 2 mL distilled water. Under vertexing at 400 rpm, 44 mg MnP, 1 mL PVA (3%, w/v), and 200 μL sodium tetraborate decahydrate (BA, 3%, w/v) were sequentially added to the DOP solution. The solution was subjected to two freeze-and-thaw cycles (-20°C for 12 h and room temperature for 8 h applied) to promote the internal cross-linking of the gel. FTIR spectrum (Gary 630, Agilent, California, USA) was recorded at room temperature. DOP-Gel hydrogels were characterized by mixing the samples with KBr powder by grinding and then compressing the mixture.

### Swelling property of DOP-Gel

The swelling ratio (SR) was determined as the weight ratio of the fully swollen gels to dry gels. The weight of lyophilized gel was defined as m_0_. The gel was immersed in water for swelling till a constant weight (m_t_) was obtained. SR was calculated as SR = (m_t_-m_0_)/m_0_×100%.

### Cytotoxicity assessment of MnP and DOP-Gel

DOP-Gel (1 mL) was added to 5 mL DMEM and incubated for 24 h. The leachates from the gel were collected and diluted with fresh medium into a series of concentrations (100%, 50%, 25%, 12.5%, 6.25%, 3.13%, 1.56%, and 0.78%), which were applied on HUVECs and B16-F10 cells pre-seeded in 96-well plates. To evaluate the cytotoxicity of MnP, the microspheres were directly added to the culture medium at various concentrations (4, 8, 15.9, 31.8, 63.5, 127, and 254 μM). The cell viability of DOP-Gel leachates or MnP was measured by a standard MTT assay after 24 h culture.

### *In vitro* ROS-responsiveness and *in vivo* release assessment of MnP@DOP-Gel

MnP@DOP-Gel (1 mL) was immersed in 10 mL PBS (pH 7.4) at different concentrations of H_2_O_2_ (0.01 mM, 0.1 mM, and 1 mM). At predetermined time points, 1 mL of supernatant was sampled and Mn^2+^ concentration was detected by ICP-MS. The cumulatively released Mn^2+^ was plotted against the time to depict the release kinetics of MnP@DOP-Gel.

*In vivo*, the degradation of MnP@DOP-Gel was assessed by subcutaneous implantation of 100 μL MnP@DOP-Gel into normal C57BL/6 mice. On days 1, 3, 6, and 10 post-implantation, two mice were sacrificed at each time point, and their skins were incised to observe the morphologies of the residual gels. In a separate set of experiments involving tumor-bearing C57BL/6 mice, a subcutaneously grafted tumor of B16-F10 was removed by resection, and then 100 μL MnP@DOP-Gel was implanted into the surgical cavity. To track the release profile of the encapsulated drugs from the DOP-Gel, Nile red-loaded MnP was used for *in vivo* imaging by an IVIS Lumina system (Perkin Elmer, Waltham, USA). Three mice were euthanized at each time point (2, 4, 6, and 8 days) after implantation, and the remaining gel was withdrawn and digested by concentrated nitric acid (68%). Mn^2+^ concentration in the gel and the blood were measured by ICP-MS.

### *In vitro* antitumor therapy

#### Cell apoptosis assay

B16-F10 cells (5 × 10^4^ per well) were seeded in 12-well plates and cultured in DMEM (supplemented with 10% FBS, 100 μg/mL penicillin, and 100 IU/mL streptomycin) medium for 24 h at 37°C with 5% CO_2_. The cell medium was then replaced with fresh medium containing PBS, MnCl_2_ (200 μM), or MnP (200 μM, 400 μM) and further incubated for 24 h. Annexin V-FITC/PI Apoptosis Detection Kit was used for cell staining according to the manufacturer's protocol. Cell apoptosis was analyzed by a flow cytometer (CytoFLEX, Beckman Coulter, Brea, USA).

#### *In vitro* ICD assessment

Two biomarkers of ICD, CRT, and HMGB1 were assessed by immunofluorescence and flow cytometry assay. B16-F10 cells (5 × 10^4^ per well) were seeded in a 12-well plate and were allowed to grow for 24 h. The cells were incubated with PBS, MnCl_2_ (200 μM), or an equivalent amount of MnP for 24 h. Cells were fixed with 4% paraformaldehyde and permeabilized with 1 mL of 0.1% Triton X-100 (in PBS) for 10 min, followed by sequential incubation with primary and secondary antibodies. Finally, after nuclei staining by DAPI, the cells were observed using a confocal laser scanning microscope (CLSM, FV3000, OLYMPUS, Tokyo, Japan). For flow cytometry, cells were first harvested by trypsin digestion and washing, followed by incubation with PE-anti-mouse HMGB1 antibody (for membrane HMGB1), or rabbit anti-CRT + FlTC goat anti-rabbit lgG(H+L) (for CRT). Finally, flow cytometry was performed.

#### cGAS-STING pathway assay of MnP-treated B16-F10 cells

cGAS-STING pathway-related markers, including cGAS, STING, IRF3, and TBK1, in MnP-treated B16-F10 cells were assessed by RT-qPCR and WB assays. B16-F10 cells were inoculated in 12-well plates at a density of 1 × 10^5^ per well and then incubated with MnCl_2_ (200 µM) or MnP (200 µM, 300 µM, and 400 µM) for 24 h. The relative expression of cGAS-STING pathway-related markers was determined using RT-qPCR, with specific primers listed in Table S2. The total RNA of the cells was extracted and then used as templates to prepare cDNAs following the instructions of the synthesis SuperMix kit. The obtained cDNAs were then amplified with the qPCR SYBR® Green Master Mix and the specific primers, and the relative mRNA levels of the target proteins were calculated with GAPDH as the internal control. For the WB assay, cells were pretreated with RIPA lysis buffer to obtain the cell lysates, and then the samples were adjusted to have equal concentrations of total proteins based on a BCA test. Each sample (equal to 10 μg total proteins) was loaded on SDS-PAGE and subjected to electrophoresis under 90-120V. Subsequently, the proteins were transferred onto polyvinylidene difluoride (PVDF) membranes with a pore size of 0.45 μm under transfer conditions of 25 V and 1 A for 30 min. Specific primary antibodies against cGAS, STING, and TBK1 were utilized, followed by overnight incubation, and then incubated with secondary antibodies (goat anti-rabbit) for 1 h. Blotting membranes were blocked using a blocking solution. Detection was performed using enhanced chemiluminescence (iBright 1500, Thermo Fisher, Waltham, USA), and the intensity of the bands was quantitatively compared between the treated groups and internal controls by ImageJ.

#### Macrophage phenotype assessment

Femurs and tibiae of C57BL/6 mice at 6-8 weeks of age were rinsed with PBS to collect bone marrow cells. The collected precursor cells were cultured in 12-well plates at a density of 1 × 10^5^ per well to induce M1 or M2 differentiation following a standard protocol 53, 54. The primary BMDMs were treated with the medium containing MnCl_2_ (200 μM) or MnP (200 μM, 300 μM, and 400 μM) and mIL-4 (40 ng/mL) for 24 h. The cells were then collected and stained with FITC-anti-mouse F4/80 antibody and APC-anti-mouse CD86 antibody.

The relative expression of M1-type cytokines including TNF-α and IFN-1β was assessed by the RT-qPCR method. BMDM cultured in 12-well plates were treated with MnCl_2_ (200 μM) or MnP (200 μM, 400 μM) and mIL-4 (40 ng/ml) for 24 h. Following treatment, the cells were collected and TNF-α and IFN-1β were determined by RT-qPCR (The specific primers used for each gene are shown in Table S2).

#### *In vitro* DC activation

Bone marrow cells were collected from the femur and tibia of mice. The collected precursor cells were cultured in DMEM (supplemented with 10% FBS) containing 20 ng/mL recombinant mouse GM-CSF and 10 ng/mL mIL-4 to induce BMDCs 55, 56. The BMDCs were incubated with MnCl_2_ (200 μM) or serial concentrations of MnP (200 μM, 300 μM, and 400 μM) for 24 h. After incubation, the cell culture supernatants were collected by centrifugation (3,000 rpm, 5 min) for assays. The sedimentary cells were collected and stained by an antibody cocktail containing FITC-anti-mouse CD11c antibody, APC-anti-mouse CD86 antibody, and PE-anti-mouse CD80 antibody for flow cytometry.

#### *In vitro* tumor inhibition of supernatant from MnP-treated BMDCs

B16-F10 cells were seeded into a 96-well plate for 24 h. The culture medium was replaced with the conditional mediums composed of ascending proportions (0, 1.6, 3.1, 6.3, 12.5, 25, 50, and 100%) of the supernatants obtained from MnP (200 μM) treated BMDCs as mentioned above. After 24 h of treatment, the cell viability was measured by MTT assay.

#### cGAS-STING pathway assay of MnP-treated BMDCs

cGAS-STING pathway-related markers, including cGAS, STING, IRF3, and TBK1, in MnP-treated BMDCs were assessed by RT-qPCR. BMDCs were inoculated in 12-well plates at a density of 1 × 10^5^ per well and then incubated with MnCl_2_ or MnP (200 µM) for 24 h. The relative expression of cGAS-STING pathway-related markers was determined by RT-qPCR (The specific primers used for each gene are shown in Table S2).

#### T cell-priming potential of the supernatants from MnP-treated B16-F10-BMDC cocultures

B16-F10 cells were firstly incubated with MnCl_2_ (200 μM) or MnP (100 μM, 200 µM, 300 μM, and 400 μM) in a 6-well plate for 24 h. The BMDCs precoated on the glass slides were added in the wells of B16-F10 cells and further incubated for 24 h to stimulate DC cell maturation. The glass slides with BMDCs were moved to a new plate, and spleen T cells were incubated with BMDCs for 48 h for T cell priming. After that, T cells were collected and added to a tumor cell precoated 96-well plate at a ratio of 10:1 (T cells: tumor cells). B16-F10 cells and LLC cells were used and inoculated in 96-well plates at a density of 2,000 cells per well. T cells and tumor cells were co-incubated for 24 h, and the viability of B16-F10 or LLC cells was then determined by LDH Cytotoxicity Assay.

### *In vivo* therapeutic assessment of MnP@DOP-Gel in post-surgical treatment of melanoma

Subcutaneous graft melanoma was established by inoculating B16-F10 cells (5 × 10^5^ cells per mouse) on the right side of the back C57BL/6 mouse (female, 6-8 weeks old). The mice were randomly divided into five groups (Control, DOP-Gel-P, Mn@DOP-Gel, MnP@Gel, and MnP@DOP-Gel). When the tumor grew to a size of about 200 mm^3^, surgical resections were carried out on the mice, with 10% of the tumor load reserved to mimic the clinical residual lesions. MnP@DOP-Gel (100 μL, at an Mn^2+^ dose of 30 mg/kg) was implanted at the excision foci of the mice. In other groups, PBS, DOP-Gel-P, Mn@DOP-Gel, and MnP@Gel were administered, respectively. Growth of the tumor residuals in the mice was monitored until reaching the experimental endpoint (the tumor threshold of 1,500 mm^3^). The TdLNs, tumor, and the major organs including the spleen, heart, liver, lung, and kidney, were excised for immune and pathological analysis (flow cytometry staining is shown in Table S3). Body weight and tumor volumes of the mice were monitored daily, and the tumor volume was calculated using the following formula: tumor volume = (tumor length) × (tumor width)^2^ × 0.5.

### Combination therapy of post-surgical implanting MnP@DOP-Gel and aPD1

The mice were divided into 6 groups, including Control, DOP-Gel-P, Free aPD1, Mn@DOP-Gel, MnP@DOP-Gel, and MnP@DOP-Gel + aPD1. The tumor inoculation, surgical resection, and post-operative implantation followed the same protocol as above. The combined aPD1 (10 mg/kg, i.p.) was administered intraperitoneally once every other day, beginning on day 0, with a total of 5 injections included in the treatment course. At the experimental endpoint (tumor size around 2,000 mm^3^), the mice were sacrificed. The TdLNs, tumors, and major organs were excised for immune and pathological analysis (flow cytometry staining is shown in Table S2). Metastatic nodules in the lungs were counted to evaluate the prophylactic effect of MnP@DOP-Gel in combination with aPD1**.**

Therapeutic potentials of MnP@DOP-Gel in combination with aPD1 were also assessed using a secondary abscopal brain tumor model. On the 5^th^ day after surgery, luciferase-labeled B16-F10 cells were intracranially inoculated in the mice (10^5^ cells in 5 μL per mouse). The growth of brain tumors was monitored by the IVIS *in vivo* imaging system using D-luciferin (150 mg/kg, i.p.) as the luminescent precursor. The survival of mice was monitored and analysed using Kaplan-Meier curves.

### Statistical analysis

Data are presented as the mean ± SD (n ≥ 3). Statistical analysis was conducted using ordinary one-way ANOVA test by GraphPad Prism. Survival analysis was conducted using Log-range (Mantel-Cox) test by GraphPad Prism. Statistical significance was indicated as *, P < 0.05; **, P < 0.01; ***, P < 0.001; and ns, nonsignificant.

## Supplementary Material

Supplementary figures and tables.

## Abbreviations

ATP: adenosine triphosphate
BA: borax
BMDCs: bone marrow-derived dendritic cells
BMDMs: bone marrow-derived macrophages
BSA: bovine serum albumin
cGAS: cyclic GMP-AMP synthase
CRT: calreticulin
DAMP: damage-associated molecular pattern
DCs: dendritic cells
DMEM: Dulbecco's modified eagle's medium
DOP: dendrobium polysaccharide
FBS: fetal bovine serum
FTIR: Fourier transform infrared spectroscopy
H&E: hematoxylin and eosin staining
HMGB1: high mobility group box-1 protein
HPIC: high performance ion chromatography
HUVECs: human umbilical vein endothelial cells
ICD: immunogenic cell death
IFNs: type I interferon
IFN-1β: interferon-1 beta
IRF3: interferon regulatory factor 3
LLC: Lewis lung carcinoma
mGM-CSF: murine granulocyte-macrophage colony-stimulating factor
mIL-4: murine interleukin-4
mM-CSF: murine macrophage colony-stimulating factor
MTT: 3-(4,5-dimethylthiazol-2-yl)-2,5-diphenyltetrazolium bromide
PBS: phosphate buffered saline
PVA: polyvinyl alcohol
ROS: reactive oxygen species
RT-qPCR: real-time quantitative polymerase chain reaction
STING: stimulator of interferon gene
SR: swelling ratio
SEM: scanning electron microscopy
TAMs: tumor-associated macrophages
TBK1: Tank binding kinase 1
TdLN: tumor-draining lymph node
TIME: tumor immunosuppressive microenvironment
TME: tumor microenvironment
Treg: regulatory T cells
WB: western blot
