# Supplementary material for: Environment-responsive dendrobium polysaccharide hydrogel embedding manganese microsphere as a post-operative adjuvant to boost cascaded immune cycle against melanoma
Source: Theranostics. 2024 Jun 17;14(10):3810–26. doi: 10.7150/thno.94354 (PMC11234272; doi:10.7150/thno.94354)
Supplement: Supplementary file 1 — Supplementary figures and tables. [file thnov14p3810s1.pdf]

Supporting information

Environment-responsive dendrobium polysaccharide hydrogel embedding manganese microsphere as a post-operative adjuvant to boost cascaded immune cycle against melanoma

Nan Gao [1, 2], Yiran Huang [1, 2], Shisuo Jing [2], Meng Zhang [3], Ergang Liu [2, *], Lu Qiu [2, 4], Jing Huang [1], Bahtiyor Muhitdinov [5], Yongzhuo Huang [1, 2, 6, 7, *]

Figure S1. Cytotoxicity of pectin, DOP, MnP, and DOP-Gel on HUVEC and B16-F10 cells.

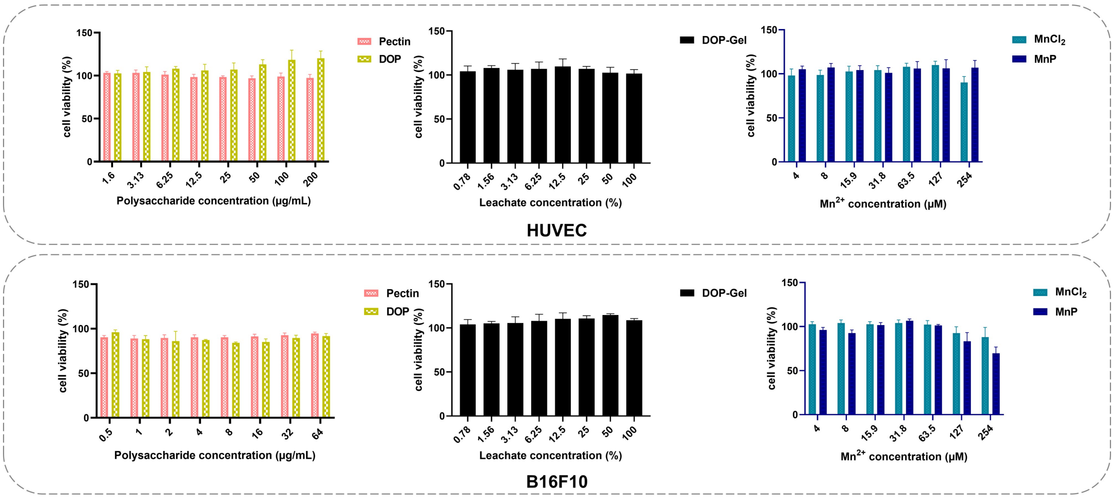

Figure S2. B16-F10 was treated with MnP ([Mn^{2+}] = 200, 300 μM) for 24 h. The protein expression levels of cGAS, TBK1, and STING were analyzed by Western blotting.

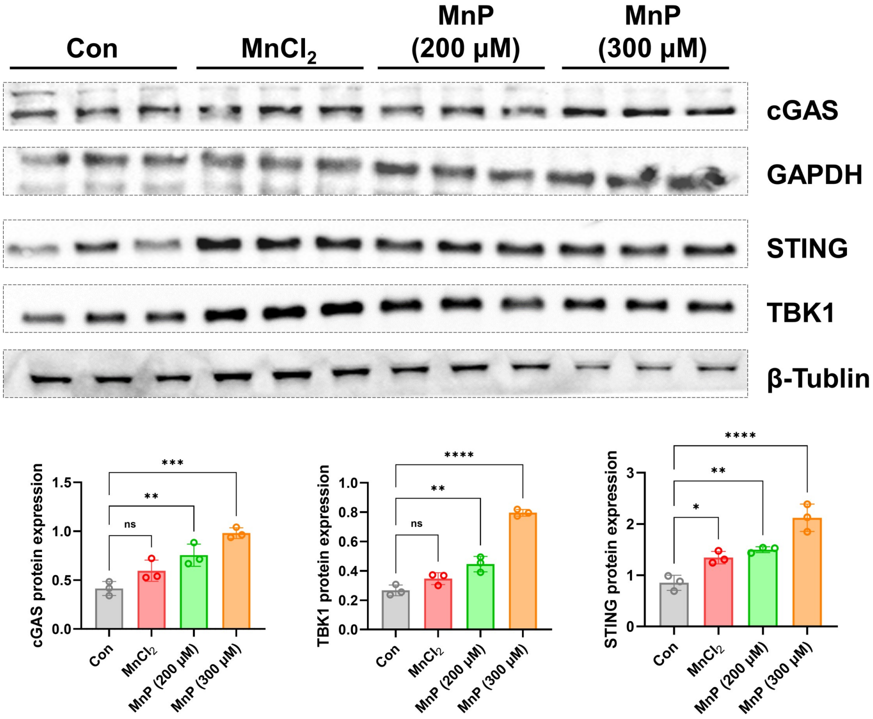

Figure S3. B16-F10 was treated with MnP ([Mn^{2+}] = 200, 300, 400 μM) for 24 h. The mRNA levels of cGAS, TBK1, IRF3, IFN-1β, and STING were analyzed by RT-qPCR

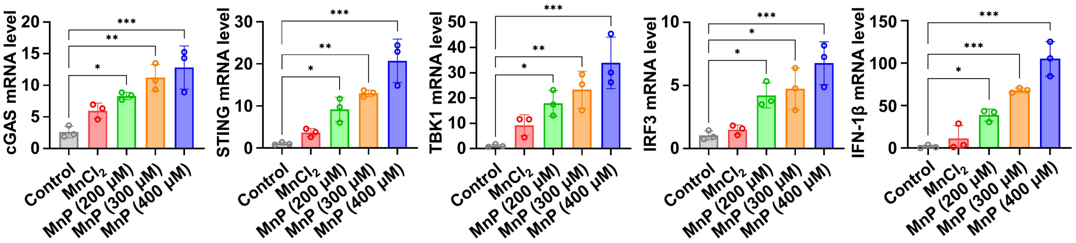

Figure S4. Fluorescence quantification of CRT and HMGB1.

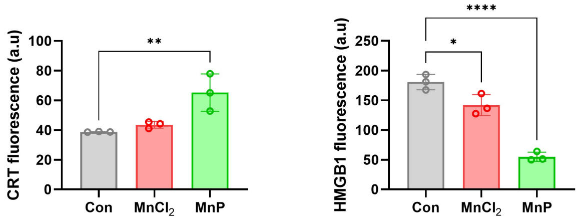

Figure S5. CRT and HMGB1 levels determined by flow cytometry.

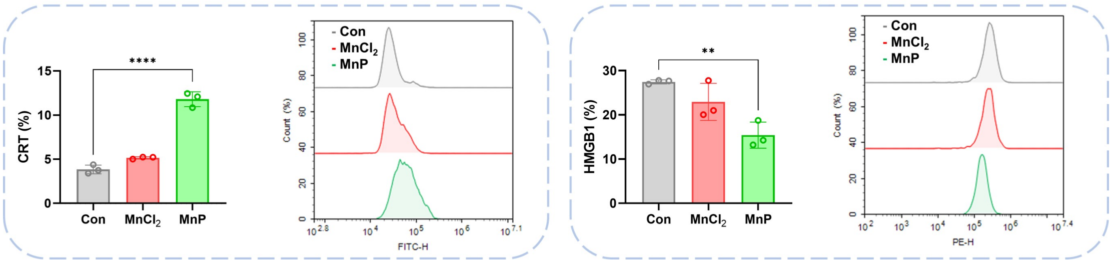

Figure S6. ATP concentration assay.

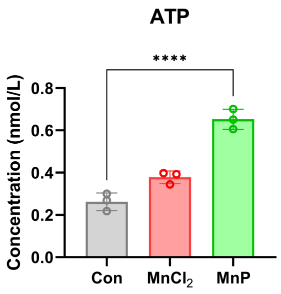

Figure S7. Representative flow cytometry plots of macrophages after MnP treatment.

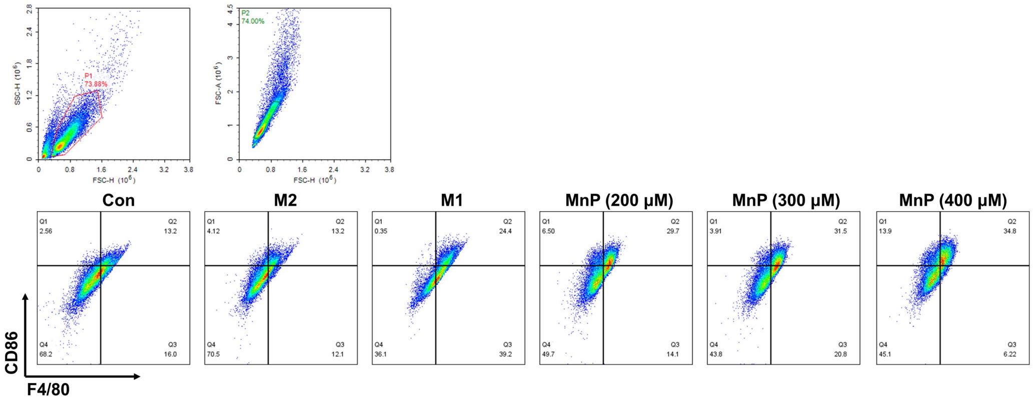

Figure S8. Cytotoxicity of Mn^{2+} on BMDC and BMDM.

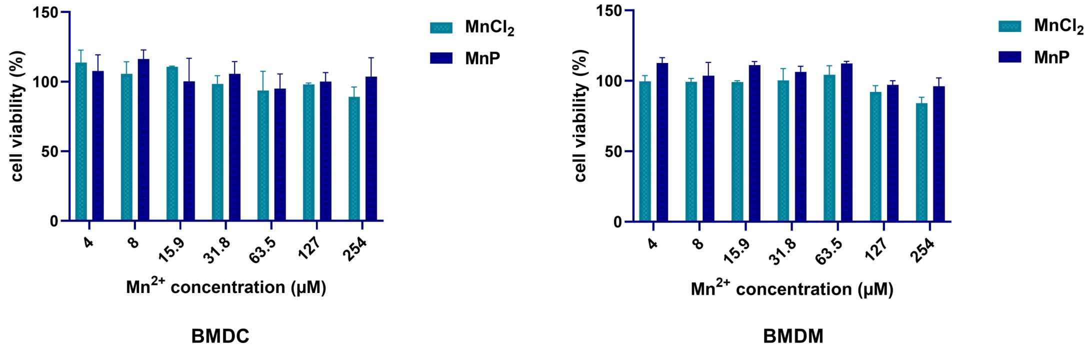

Figure S9. Representative flow cytometry plots of mature DCs after MnP treatment.

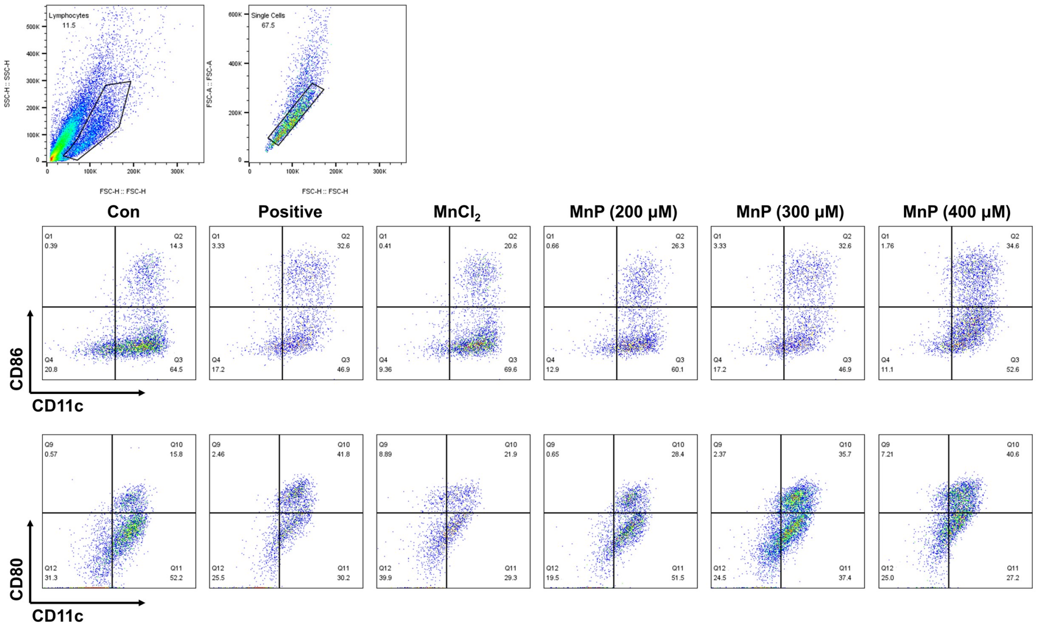

Figure S10. Flow cytometric detection of DC maturation after co-culture with MnP-treated B16F10 cells.

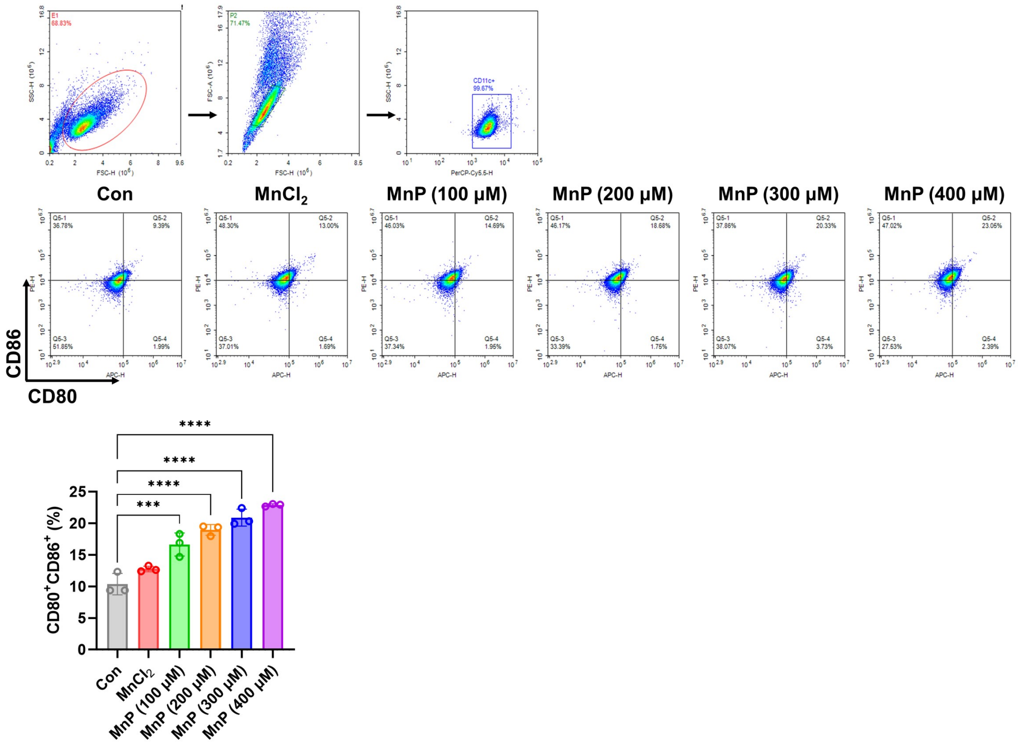

Figure S11. The viability of LLC and B16-F10 cells after co-incubation with the T cells pretreated with MnP ([Mn^{2+}] = 200 μM).

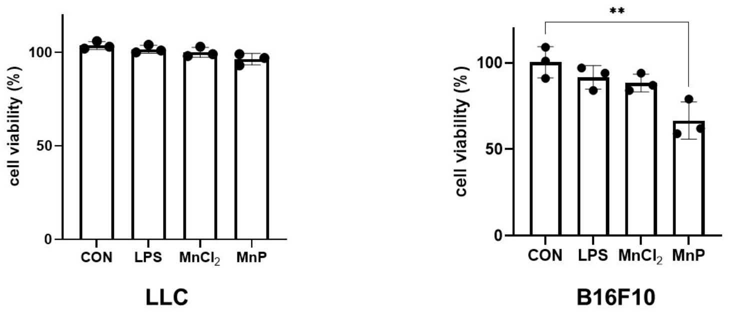

Figure S12. Tumor inhibition by implantation of MnP@DOP-Gel in a subcutaneous B16-F10 tumor model.

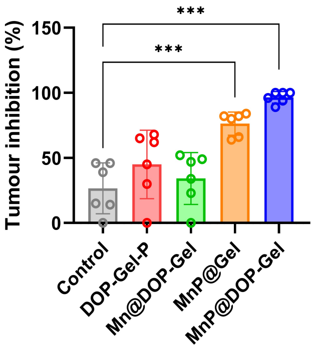

Figure S13. Representative flow cytometry plots of M1-like macrophages in the inguinal lymph nodes and spleen.

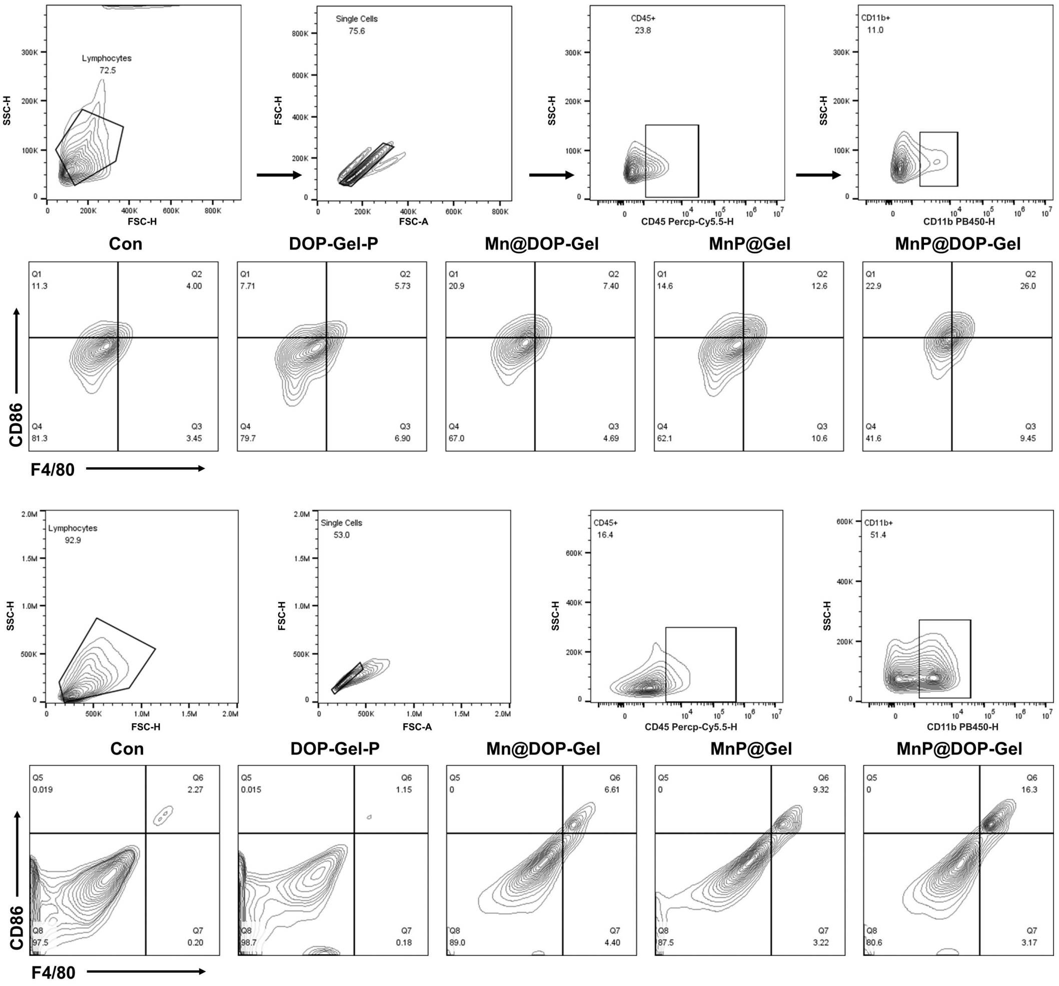

Figure S14. Representative flow cytometry plots of CD8$^+$ T and CD4$^+$ T cells in the spleen and reoccurring tumors.

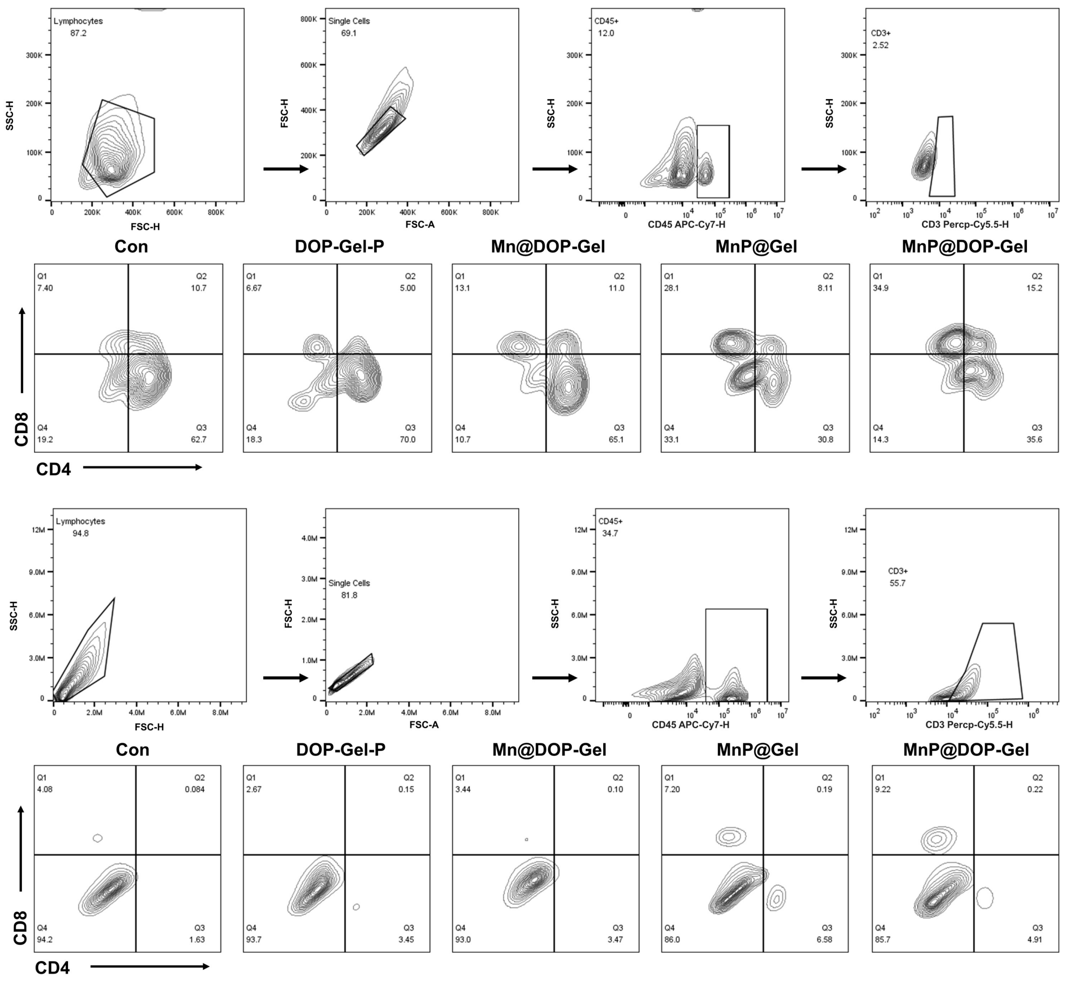

Figure S15. Body weight changes of the mice during the experiment.

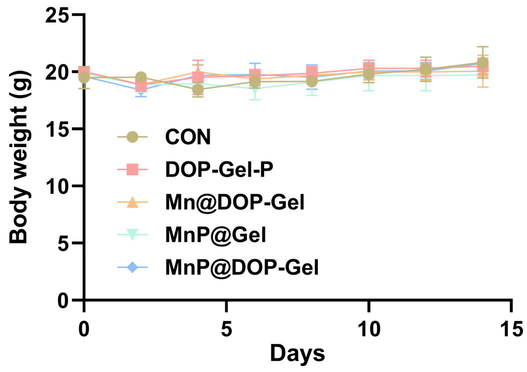

Figure S16. Representative H&E images of the heart, kidney, and spleen.

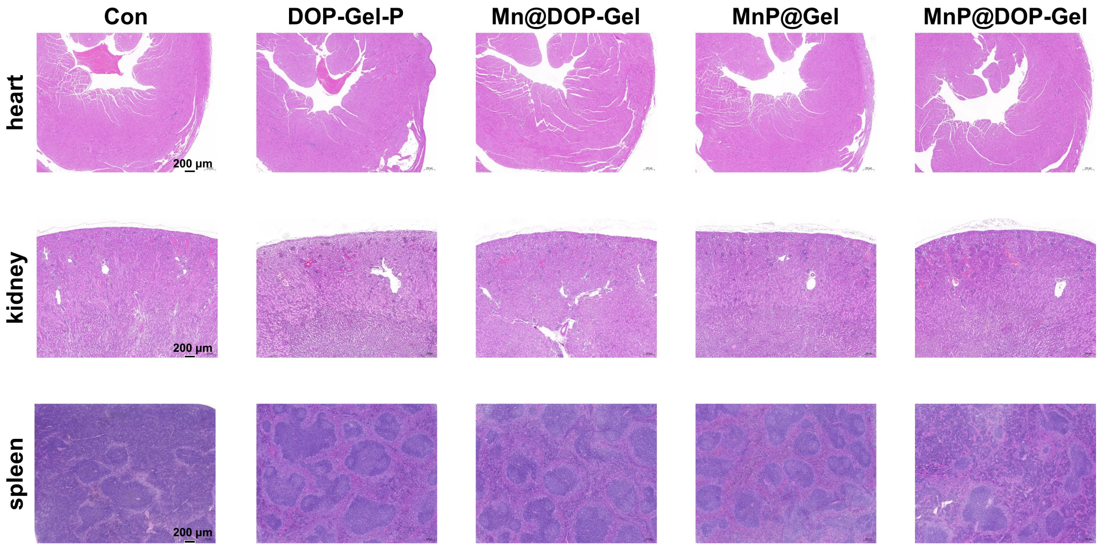

Figure S17. Tumor inhibition rate of combination therapy of MnP@DOP-Gel and aPD1.

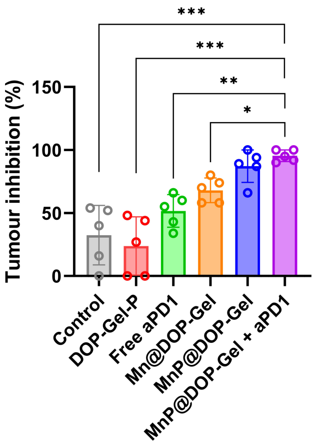

Figure S18. Representative flow cytometry plots of mature DCs in the reoccurring tumors and lymph nodes.

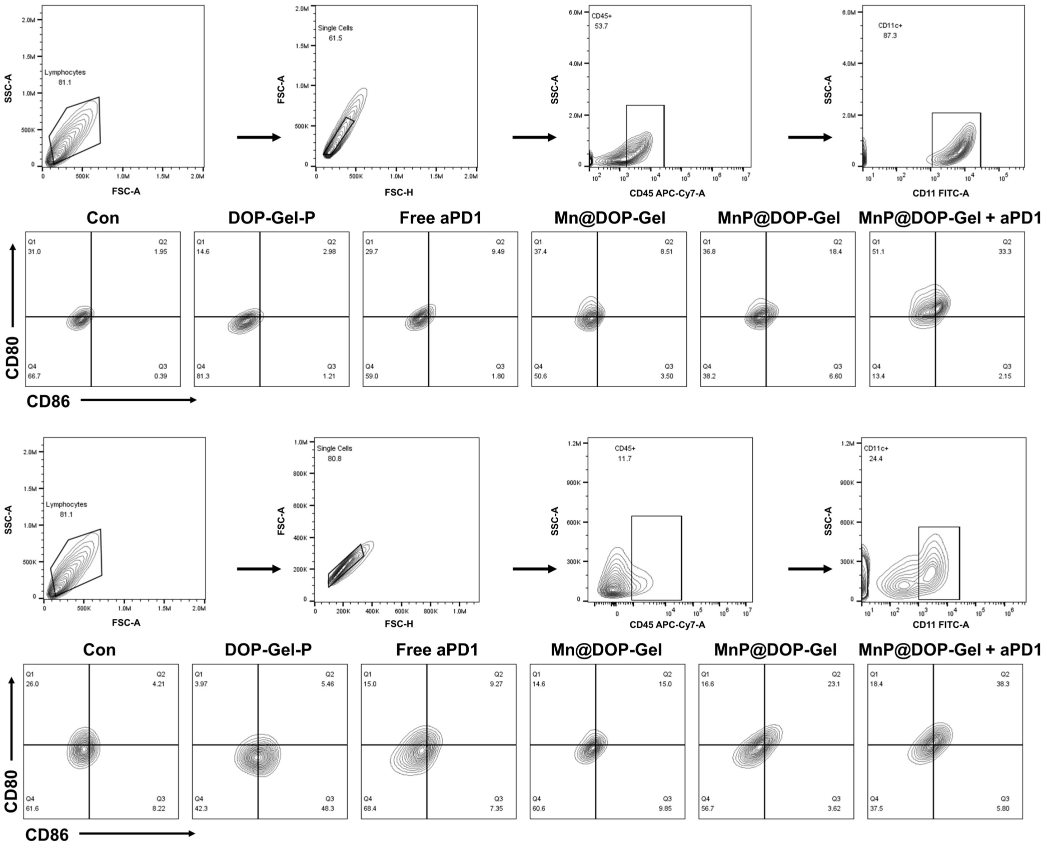

Figure S19. Representative flow cytometry plots of cytotoxic T cells and regulatory T cells in the spleen.

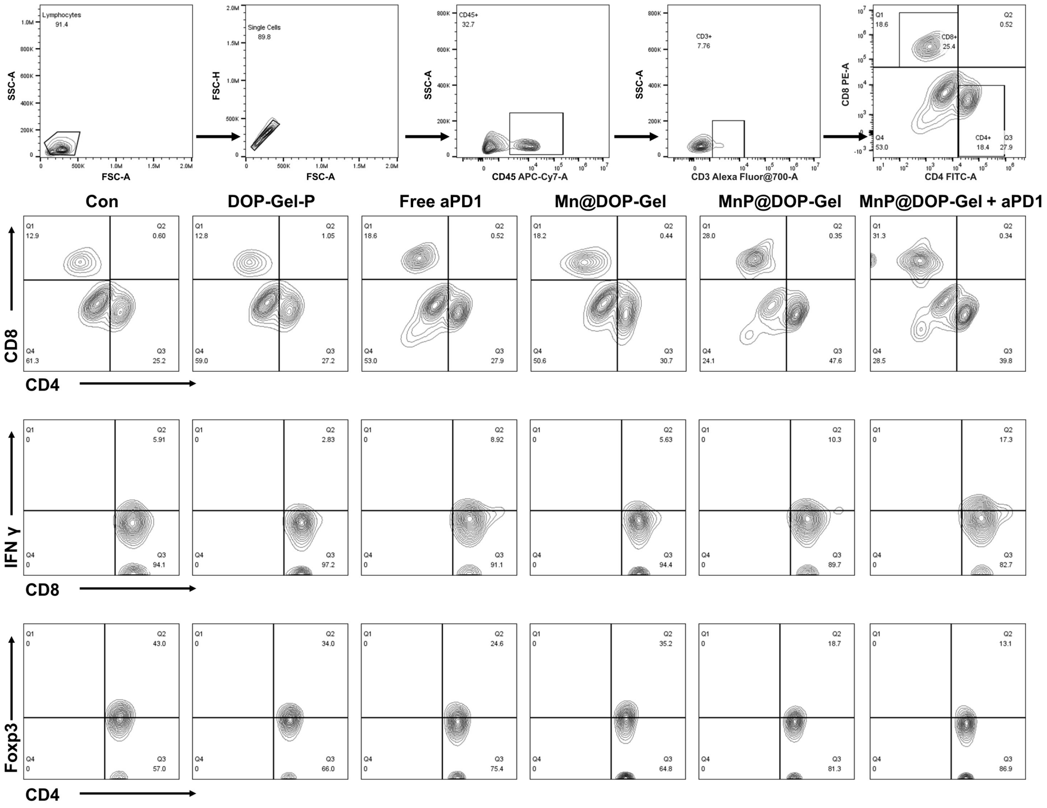

Figure S20. Ki67 fluorescence quantification.

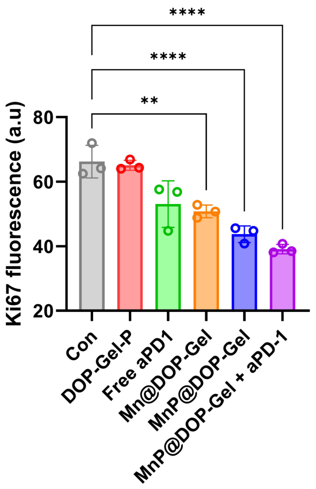

Figure S21. Fluorescence quantification of IRF3, cGAS, and STING in the reoccurring tumors.

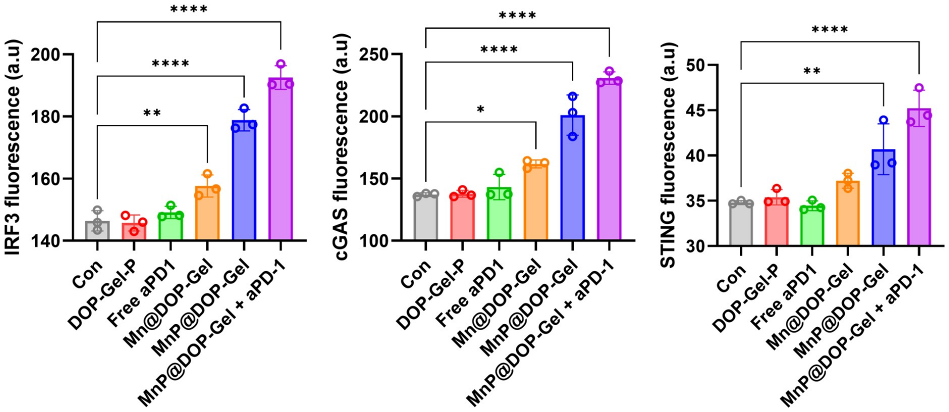

Table S1. Serum manganese levels after post-operative MnP@DOP-Gel implantation.

Groups	Mn (S-SQ-KED)			
	2 d	4 d	6 d	8 d
Con (n = 3)	3073.07±435.24	3085.40±428.77	3337.10±305.90	2865.37±920.67
MnP@DOP-Gel (n = 3)	3117.40±372.91	3087.37±546.60	3085.07±461.10	3267.40±80.68

* Note: no statistical difference in blood Mn concentration between the tested group and the normal control group. Due to the values below the limit of quantification (Mn, 1 μg/ml) of the S-SQ-KED method, raw data are listed in the table.

Table S2. Primer sequences for real-time PCR assays.

Gene	Primer
TNFA-F	CTGTGAAGGGAATGGGTGTT
TNFA-R	CAGGGAAGAATCTGGAAAGGTC
IFN-B-F	CACAGCCCTCTCCATCAACT
IFN-B-R	TCCCACGTCAATCTTTCCTC
STING-F	TGTTGCTGTAAACCCGATC
STING-R	ATAACCTGAGTATGGCTGACC
TBK1 F-2	GGAGCCGTCCAATGCGTAT
TBK1 R-2	GCCGTTCTCTCGGAGATGATTC
IRF3 F-2	GAGAGCCGAACGAGGTTCAG
IRF3 R-2	CTTCCAGGTTGACACGTCCG
GAPDH F	TGATGGGTGTGAACCACGAG
GAPDH R	TAGGGCCTCTCTTGCTCAGT
cGAS F	AGGAAGCCCTGCTGTAACACTTCT
cGAS R	AGCCAGCCTTGAATAGGTAGTCCT
β-Act-F	TGTCCACCTTCCAGCAGATGT
β-Act-R	AGCTCAGTAACAGTCCGCCT

Table S3. Antibodies and dyes used in flow cytometry.

Cell subpopulation	Tissue source	Flow antibody
$CD4^+$ and $CD8^+$ T cell	spleen	anti-CD45-Alexa Fluor@700
	tumor	anti-CD3-Percp-Cy5.5
		anti-CD4-APC
		anti-CD8-PE
M1-type Macrophage	spleen	anti-CD45-Percp-Cy5.5
	lymph node	anti-CD11b-Brilliant Violet 421
		anti-F4/80-FITC
		anti-CD86-PE-CY7
Functional T cell	lymph node	anti-CD45-APC-Cy7
	tumor	anti-CD3-Percp-Cy5.5
		anti-CD4-FITC
		anti-CD8-PE
		anti-IFN-γ-Brilliant Violet 510
		anti-Foxp3-Brilliant Violet 421
DCs	lymph node	anti-CD45-APC-Cy7
	tumor	anti-CD11c-FITC
		anti-CD80-PE
		anti-CD86-APC
